# High levels of species' extirpation in an urban environment—A case study from Berlin, Germany, covering 1700–2023

**DOI:** 10.1002/ece3.70018

**Published:** 2024-07-15

**Authors:** Silvia Keinath, Shenya De Silva, Nike Sommerwerk, Jörg Freyhof

**Affiliations:** ^1^ Museum für Naturkunde Berlin–Leibniz Institute for Evolution and Biodiversity Science Berlin Germany

**Keywords:** centuries, extinction, habitats, Red Lists, taxonomic groups, urbanisation

## Abstract

Species loss is highly scale‐dependent, following the species–area relationship. We analysed spatio‐temporal patterns of species' extirpation on a multitaxonomic level using Berlin, the capital city of Germany. Berlin is one of the largest cities in Europe and has experienced a strong urbanisation trend since the late nineteenth century. We expected species' extirpation to be exceptionally high due to the long history of urbanisation. Analysing 37 regional Red Lists of Threatened Plants, Animals and Fungi of Berlin (covering 9498 species), we found that 16% of species were extirpated, a rate 5.9 times higher than at the German scale and 47.1 times higher than at the European scale. Species' extirpation in Berlin is comparable to that of another German city with a similarly broad taxonomic coverage, but much higher than in regional areas with less human impact. The documentation of species' extirpation started in the eighteenth century and is well documented for the nineteenth and twentieth centuries. We found an average annual extirpation of 3.6 species in the nineteenth century, 9.6 species in the twentieth century and the same number of extirpated species as in the nineteenth century were documented in the twenty‐first century, despite the much shorter time period. Our results showed that species' extirpation is higher at small than on large spatial scales, and might be negatively influenced by urbanisation, with different effects on different taxonomic groups and habitats. Over time, we found that species' extirpation is highest during periods of high human alterations and is negatively affected by the number of people living in the city. But, there is still a lack of data to decouple the size of the area and the human impact of urbanisation. However, cities might be suitable systems for studying species' extirpation processes due to their small scale and human impact.

## Graphical abstract

Our study compiled and analysed spatio‐temporal species' extirpation on a broad taxonomic coverage at the city scale. We revealed high number of extirpations, which are in the twenty‐firstcentury already comparable with previous centuries, and showed that cities might be suitable systems for studying species' extirpation processes due to their small scale and human impact.

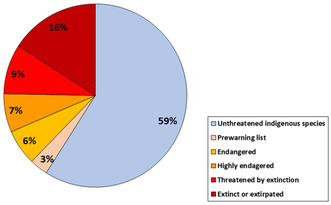

## 1 INTRODUCTION

Global change, caused by human intervention in the environment, is affecting the Earth's ecosystems, leading to widespread changes in biodiversity (Chapin et al., 2000; Steffen et al., 2011). Although species loss is a natural process driven by evolution (Raup, 1994; Wiens et al., 2012), its exaggeration is one of the consequences of human‐induced environmental change, mostly driven by habitat loss and fragmentation, overexploitation, invasive alien species, disease and climate change (Purvis et al., 2000; Roy et al., 2023; Sodhi et al., 2009). ‘Species loss’ is usually a gradual process and we distinguish between species' extirpation, defined as the local disappearance of species as a gradual loss of populations, and species' extinction, defined the global, irreversible death of species when the last individual has died (Smith‐Patten et al., 2015). Species' extinction is a selective process, when their adaptations no longer fit their environment (Jablonski, 1986; Purvis et al., 2000), starting with negative trends in species' abundance, followed by a decline in species' distributions (extirpation), and become threatened by extinction when species' population sizes drop below the threshold of viability (Turnhout & Purvis, 2020). This process starts at a small scale and, when spreading to larger scales, the long‐term consequences might be global extinction (Purvis et al., 2000; Sodhi et al., 2009). Species with small ranges, high habitat specificity, low population densities and/or low reproductive rates are particularly vulnerable to the process of extirpation (Sodhi et al., 2009).

While there is a common agreement that we have entered the sixth mass extinction (Nazarevich, 2015; Wollmuth et al., 2022), global, continental and national extinction levels of known species are still low. ‘Only’ 0.76% of all species, considering the 128,918 species that are listed in the IUCN Red List of Threatened Species, are actually listed as globally extinct or extinct in the wild (when still alive in captivity). When the ‘possibly extinct’ or ‘possibly extinct in the wild’ species are also considered, the value totals 1.78% (IUCN Summary Statistics, 2023). At the European scale, Hochkirch et al. (2023) found that ‘only’ 0.34% of the 14,669 species (representing about 10% of Europe's fauna and flora) are extinct, extinct in the wild or extirpated. In Germany, 2.7% of the 32,354 species assessed in the national Red Lists of Threatened Plants, Animals and Fungi are considered extirpated, among them are ‘only’ three globally extinct freshwater fish species.

Species loss is highly scale‐dependent, as smaller areas have, on average, less diverse habitats and host fewer species, increasing the probability of species' extirpation. This process is described by the species–area relationship (Halley et al., 2013). Therefore, extirpation levels should be much higher at local scales, especially in areas that have experienced a long history of habitat alteration and other threats. This situation has been well studied on islands (Cooke et al., 2023; Lewis, 2018) and lakes (Witte et al., 1992). If species are additionally endemic to these local areas, their extirpation process might rapidly lead to extinctions.

Other small‐scale areas are cities. Urban areas also have a long history of human influence in terms of habitat alterations. However, cities are unlike islands, as their edges are open to species invasion and retreat. They are therefore not expected to have high number of endemic species. However, cities might be suitable model systems for studying species loss through extirpation, as high levels of urbanisation lead to strong derivations of ecosystems from their ‘natural’ conditions. Furthermore, urbanisation is a common global situation and it is one of the major, steadily increasing interventions to the environment worldwide (Goddard et al., 2010; Liu et al., 2016; Soundranayagam et al., 2011). By 2050, 68% of the world's human population is projected to live in cities, an increase of 38% since 1950 (United Nations, 2019, 2022). In 2022, already 77.7% of the German human population live in cities (Statista, 2024a) and it is predicted that nearly 85% will be living in cities by 2025 (Statista, 2024b). This rapid growth of urban areas might play an important role in addressing the global extinction crisis (Knapp et al., 2021), as the consequences of urbanisation, such as resource demands, pollution and climate impacts affect ecosystems far beyond urban areas (McDonald et al., 2020).

The consequences of urbanisation on biodiversity are multifaceted (Aronson et al., 2017; McKinney, 2008; Seto et al., 2012): The high density of human settlements in cities leads to spatial interventions, such as fragmentation, and loss of habitats for built‐up areas, industry and infrastructure, such as railways and roads (Czech et al., 2000; Goddard et al., 2010; Liu et al., 2016; Marzluff, 2001). Urbanisation is also associated with air pollution from traffic and industrial emissions (Fenger, 1999), light pollution (Falchi, 2019), Urban Heat Islands due to surface sealing and high building density (Rizwan et al., 2008) and the introduction of invasive alien species (Doherty et al., 2016; Pyšek et al., 2020). These factors interact as threats to local biodiversity (McDonald et al., 2008; McKinney, 2002; Theodorous, 2022). However, cities also exhibit heterogeneous habitat structures at small scales (Kühn et al., 2004). Even within densely urbanised areas, there are recreational greenspaces, private gardens, railway and roadside greenspaces, rivers and lakes, greenspaces in industrial areas, small or large leftover areas, urban rewilding areas at ‘lost places’ and many more. Thus, urban areas are not homogeneous and provide very diverse habitats for biodiversity, shaping very specific urban novel ecosystems (Haaland & van den Bosch, 2015; Niemeier et al., 2020), especially when compared to agricultural areas (Kühn et al., 2004). Urban novel ecosystems are mostly not inhabited by the same biological community that had originally inhabited the site prior to urbanisation (Hobbs et al., 2013; McIntyre, 2000). However, indigenous species, that persist throughout the urbanisation process may adapt to novel urban ecosystems (Doudna & Danielson, 2015; Keinath et al., 2020, 2021, 2023; Niemeier et al., 2020; Van't Hof et al., 2011), and co‐occur with those species that invade urban areas after the habitat alternation provides them with suitable ecological conditions. Other species, on the other hand, are not able to cope with these changes and are extirpated from urban areas (Hahs et al., 2009; Magle et al., 2010).

Following the predictions from the species–area relationship (small areas are likely to have high species shifts) and the high level of anthropogenic alterations (many species are lost due to anthropogenic impacts), species' extirpation in cities are expected to be exceptionally high. This has very rarely been studied for more than a few selected taxonomic groups. Thus, most studies examining species' extirpation in urban areas are taxonomically biased by focusing on individual taxonomic groups, such as plants (Bertin, 2002; Colling, 2005; Duncan et al., 2011; Gregor et al., 2012; Hahs et al., 2009; Williams et al., 2005), birds (Dri et al., 2021) and individual insect groups (Fattorini, 2011; Theng et al., 2020). Studies examining species' extirpations in habitats and regions outside urban areas are also taxonomically biased (Bonebrake & Cooper, 2014; Fattorini, 2020; Maes & Van Dyck, 2001; Suhonen et al., 2014). These studies also cover very different spatio‐temporal scales, making it difficult to compare levels of extirpation between urban and rural areas. By compiling data from 18 European countries and regions, Colling (2005) showed that the proportion of extirpated vascular plant species decreased significantly with increasing area surveyed. Furthermore, Williams et al. (2005) showed that extirpation rates of grassland plants were higher in urban (37%) than in suburban (27%) and rural (20%) areas in western Victoria, Australia. At a multitaxonomic level on regional scales, we found only unpublished overview analyses of the Red Lists of Threatened Plants, Animals and Fungi from different German federal states. They showed that the percentage of extirpated species is highest in the city of Hamburg (755 km^2^; Behörde für Umwelt, Klima, Energie und Agrarwirtschaft Hamburg, 2024; Hamburg in Zahlen, 2024) with 15%, compared to the rural‐dominated federal states of Saarland (2570 km^2^), which consists of more than 50% agricultural landscapes and has 6.2% species' extirpation (Rote Liste Saarland, 2024; Saarland, 2022), and Niedersachsen (47,614 km^2^) with 5.8% species' extirpation (Niedersachsen, 2024; NLWKN, 2024).

In our study, we examined species' extirpation at a multitaxonomic level at the city scale in space and time, using the German capital, Berlin, as a case study. As Berlin is highly urbanised and has a long history of urbanisation, we expected the percentage of extirpated species not only to be higher at this city scale compared to the German or European scales, but also to be much higher compared to rural, less urbanised areas. On a temporal scale, we predicted species' extirpation to be highest in the last and most recent centuries, when urbanisation was at its highest level so far. We also predicted species' extirpation to correlate with the number of inhabitants in the city over time, due to increasing land use and urban densification.

## 2 MATERIALS AND METHODS

### 2.1 Study area

Berlin (52°31′ N, 13°24′ E), the federal city‐state, capital and largest city of Germany, was founded in the thirteenth century. Since the Industrial Revolution in the 1800s, Berlin's human population has grown rapidly (Ribbe et al., 2002a; Ring, 1992) due to rapid industrialisation (Ribbe et al., 2002a, 2002b; Wey, 1982). During this period, Berlin was affected by high levels of environmental pollution, such as air pollution and the application of large quantities of chemical fertilisers in the cities' surroundings (Erisman et al., 2008; Le, 2008; Pamme, 2003; Ramankutty et al., 2018; Wey, 1982). The city passed the 1 million population mark in 1877 and was expanded to ‘Groß‐Berlin’ in the 1920s with an increase of 66 km^2^ (Statista, 2024c). This led to rapid construction of buildings and infrastructure, reaching a maximum of 4.3 million inhabitants before World War II (Buesch & Haus, 1987). After the Second World War in 1945, Berlin's population had shrunk to 3 million inhabitants and then slowly increased again (Ring, 1992). In the 1950s, the first environmental protection measures were implemented to reduce air pollution (Pamme, 2003; UBA, 1998; UNEP/WHO, 1993) and to reduce the use of chemical fertilisers in agriculture in the surroundings of the city (Erisman et al., 2008; Tilman et al., 2002). During the post‐war reconstruction of the heavily destroyed city, Berlin was divided into a western and an eastern part and finally separated by the Berlin Wall between 1961 and 1989 (Ribbe et al., 2002b; Schildt & Sywottek, 1993). Since German reunification in 1990, Berlin has gained more than 300,000 new residents, an increase of 9% (Bund‐Länder Demografie Portal, 2024). Between 2018 and 2021, Berlin's population stagnated at nearly 3.7 million, but in 2023, it reached 3.87 million and a population density of 4.34 inhabitants per km^2^ (Statista, 2023), the highest level since the 1920s. Today, Berlin is one of the greenest and, at the same time, most densely populated cities in Europe (Schewenius et al., 2014). The city covers an area of 892 km^2^ and is covered by approximately 59% built‐up areas, 35% greenspaces, including forests, parks, allotment gardens, fields and meadows, as well as 6% blue spaces, such as lakes and rivers. Berlin has 44 protected natural areas, covering 2.73 ha and 3.1% of the Berlin area (SenStadt, 2020). A systematic and standardised survey of the proportion of sealed surfaces, open spaces, residential areas and transportation areas for the whole of Berlin has only been carried out since the 1990s (Umweltatlas Berlin, 2020, 2021) due to the division of the city (Ribbe et al., 2002b; Schildt & Sywottek, 1993).

### 2.2 Red Lists of Threatened Plants, Animals and Fungi of Berlin

While the IUCN (International Union for Conservation of Nature) Red List of Threatened Species assesses the global endangerment of species, there are Red Lists at the national scale and below, for example, at the regional scale, such as for the 16 German federal states, which refer specifically to the status of species within the respective federal state. The IUCN categories are determined using a different methodology (IUCN Standards and Petitions Committee, 2022) than the German national and regional Red Lists, which, for example, consider a longer time scale of species' population decline than the IUCN and are less focused on extinction risk. A direct comparison of the results of the global IUCN Red Lists and the national and regional Red Lists is therefore only possible to a limited extent (Rote Liste Zentrum, 2024b).

The first Red Lists of Threatened Plants, Animals and Fungi of Berlin were published in 1982, limited to the western part of the city (Sukopp & Elvers, 1982). In 1991, after the reunification of Germany, a revised version was published (Auhagen, 1991), which still focused mainly on the western part of the city. Only since 2009 have Red Lists been published for the entire city‐state of Berlin, following the standardised national German criteria (Ludwig et al., 2009; Saure & Kielhorn, 2005). The Berlin Red Lists are compiled by various experts in cooperation with the administration of the city‐state government, the Berlin Senate Department for Mobility, Transport, Climate Protection and the Environment (SenMVKU, 2024a).

Before 2009, there was a variety of poorly documented criteria, making it difficult to compare the conservation status of species over time. However, the criteria for classifying a species as being ‘extinct or extirpated in Berlin’ remained unchanged before and after 2009. The prerequisites for awarding species in category 0, that is, ‘extinct/extirpated’, are (i) the reliable evidence that the species was indigenous or firmly naturalised in Berlin (Saure & Schwarz, 2005), and (ii) despite searches, has no longer been detected, and therefore there are (iii) valid reasons for suspecting that the species' populations have all vanished. Red List experts moreover researched historical floral, faunal and fungal records from the literature and museum collections to provide additional estimates of historical species loss. However, species might have extirpated unrecorded, especially in poorly studied taxa groups. For each taxonomic group, there is a defined minimum time after which a species can be considered as being extinct/extirpated if the search is unsuccessful (Saure & Schwarz, 2005). For vertebrates, the minimum time is set at 10 years, for invertebrates at 20 years (Binot et al., 1998). For vascular plants, the minimum time is set at 10 years and for algae, mosses, lichens and fungi at 20 years (Prasse et al., 2001). The Berlin Red Lists are mainly published every 10 years (SenMVKU, 2024a).

The objectives of regional Red Lists are the protection and conservation of biodiversity, its monitoring and evaluation, planning and decision support and public relations (SenMVKU, 2024a). National and regional Red Lists are indispensable tools in the processes and procedures of nature conservation intervention regulation, in the designation of protected areas or the planning of species and biotope protection programmes. Red Lists have therefore become an important touchstone for the efficiency of nature conservation measures (Riecken et al., 2000; Schnittler et al., 1994).

### 2.3 Data extraction

To determine the proportion of extirpated species for Germany, we manually summarised the numbers of species classified in category 0 ‘extinct or extirpated’ and calculated the percentage in relation to the total number of species listed in the Red Lists of Threatened Species for Germany, taken from the website of the Red List Centre of Germany (Rote Liste Zentrum, 2024a).

For Berlin, we used the 37 current Red Lists of Threatened Plants, Animals and Fungi from the city‐state of Berlin, covering the years from 2004 to 2023, taken from the official capital city portal of the Berlin Senate Department for Mobility, Transport, Climate Protection and Environment (SenMVKU, 2024a; see overview of Berlin Red Lists used in Table 1). We extracted all species that are listed as extinct/extirpated, that is, classified in category 0, and additionally, if available, the date of the last record of the species in Berlin. The Red List of macrofungi of the order Boletales by Schmidt (2017) was not included in our study, as this Red List has only been compiled once in the frame of a pilot project and therefore lacks the category 0 ‘extinct or extirpated’.

**TABLE 1:** Overview of the 37 applied Red Lists of Threatened Plants, Animals, and Fungi from the federal state of Berlin, Germany, and pooled taxonomic groups (grey marked, bold) with number of unthreatened indigenous species, number of species in the respective Red List hazard categories [Prewarning list, endangered (category 3), highly endangered (category 2), threatened by extinction (category 1), extinct or extirpated (category 0)], total number of indigenous species, and number of non‐indigenous species (neobiota and reference).

Taxon	Unthreatened indigenous species	Prewarning list	Endangered (category 3)	Highly endangered (category 2)	Threatened by extinction (category 1)	Extinct or extirpated (category 0)	Total numbers of indigenous species	Non‐indigenous species (neobiota)	Reference
Weevils (Curculionoidea)	317	13	46	47	34	85	542	0	Bayer and Winkelmann (2005)
Capuchin beetles (Bostrichoidea), multicoloured beetles (Cleroidea), flat beetles (Cucujoidea), click beetles (Elateroidea), shipyard beetles (Lymexyloidea) and black beetles (Tenebrioidea)	519	3	38	83	24	40	707	31	Esser (2017c)
Leaf beetles (Chrysomelidae und Megalopodidae)	165	15	11	12	16	32	251	11	Heinig and Schöller (2017)
Aquatic beetles (Coleoptera: Hydradephaga, Hydrophiloidea part, Hydraenidae, Elmidae und Dryopidae)	107	9	10	21	18	31	195	0	Heinrich and Müller (2017)
Short‐winged beetles and hister beetles (Coleoptera: Staphylinoidea und Histeridae)	672	2	24	81	68	116	963	10	Esser (2017b)
Scarab beetles (Coleoptera: Scarabaeoidea)	39	1	9	10	12	22	93	0	Esser (2017a)
Long‐horned beetles (Coleoptera: Cerambycidae)	67	3	6	4	1	8	89	3	Esser (2017d)
Jewel beetles (Coleoptera: Buprestidae)	32	0	2	0	6	7	47	0	Gottwald (2017)
Ground beetles (Coleoptera: Carabidae)	184	0	24	23	23	34	288	0	Kielhorn (2005)
Cicadas (Hemiptera: Fulgoromorpha und Cicadomorpha)	225	12	15	5	25	39	321	8	Nickel and Mühlethaler (2017)
True bugs (Heteroptera)	342	12	8	13	25	88	488	7	Deckert and Burghardt (2018)
Grasshoppers and crickets (Saltatoria: Ensifera et Caelifera)	20	7	1	2	8	8	46	0	Machatzi et al. (2005)
Dragonflies (Odonata)	42	0	3	3	6	4	58	0	Petzold (2017)
Bees and wasps (Hymenoptera part.) with ants	366	55	50	50	45	98	664	0	Saure (2005b)
Mayflies (Ephemeroptera)	9	0	3	1	1	0	14	0	Müller (2017)
Earwingflies (Mecoptera)	5	0	0	0	0	0	5	0	Saure (2005c)
Robber flies (Diptera: Asilidae)	27	1	0	0	6	8	42	0	Degen (2017)
Hoverflies (Diptera: Syrphidae)	162	9	11	11	28	31	252	0	Saure (2018)
Snakeflies, alderflies, dobsonflies and lacewings (Raphidioptera, Megaloptera, Neuroptera)	59	0	10	0	0	5	74	0	Saure (2005a)
Caddisflies (Trichoptera)	69	0	16	6	10	7	108	0	Müller and Mey (2017)
Bagmoths (Lepidoptera: Psychidae)	9	0	4	1	0	5	19	0	Weidlich (2022)
Butterflies and moths (Lepidoptera: Makrolepidoptera)	530	8	64	48	56	150	856	0	Gelbrecht et al. (2022)
Spiders (Araneae) and harvestmen (Opiliones)	367	7	27	23	55	57	536	33	Kielhorn (2017)
**Arthropods**	**4334**	**157**	**382**	**444**	**467**	**876**	**6659**	**103**	
**Molluscs (Mollusca: Gastropoda und Bivalvia)**	**75**	**6**	**18**	**6**	**15**	**8**	**128**	**15**	Hackenberg and Müller (2017)
**Fish and lampreys (Pisces et Cyclostomata)**	**11**	**4**	**4**	**2**	**0**	**7**	**28**	**8**	SenMVKU (2014)
**Reptiles (Reptilia)**	**1**	**3**	**0**	**1**	**1**	**1**	**7**	**0**	Kühnel et al. (2017b)
**Amphibians (Amphibia)**	**2**	**0**	**4**	**2**	**2**	**2**	**12**	**1**	Kühnel et al. (2017a **)**
**Breeding birds (Aves)**	**75**	**11**	**17**	**6**	**17**	**32**	**158**	**7**	Witt and Steiof (2013 **)**
**Mammals (Mammalia)**	**38**	**0**	**8**	**3**	**2**	**4**	**55**	**2**	Klawitter et al. (2005)
Limnic red algae (Rhodophyta) and brown algae (Phaeophyceae)	7	0	0	0	0	1	8	0	Rudolph et al. (2017)
Stonewort algae (Characeae)	2	4	0	4	0	11	21	0	Kusber et al. (2017)
**Algae**	**9**	**4**	**0**	**4**	**0**	**12**	**29**	**0**	
Slime fungi (Myxomycetes incl. Ceratiomyxomycetes)	192	0	4	5	3	21	225	0	Schmidt and Täglich (2023)
Smut fungi (Ustilaginales)	14	8	7	0	22	44	95	0	Scholz and Scholz (2005)
Lichen‐dwelling (lichenicolen) fungi	21	0	0	0	0	2	23	0	Wagner et al. (2017)
**Fungi**	**227**	**8**	**11**	**5**	**25**	**67**	**343**	**0**	
**Lichens (Lichenes)**	**216**	**6**	**9**	**14**	**11**	**59**	**315**	**0**	Krause et al. (2017)
Mosses (Bryophyta)	187	9	39	24	46	100	405	3	Klawitter and Köstler (2017)
Ferns and flowering plants	178	82	77	100	210	266	913	307	Seitz et al. (2018)
**Plants**	**365**	**91**	**116**	**124**	**256**	**366**	**1318**	**310**	
**Total**	**5353**	**290**	**569**	**611**	**796**	**1433**	**9052**	**446**	

We used Python, version 3.7.9 (Van Rossum & Drake, 2009), the Python libraries Pandas (McKinney, 2010) and Camelot‐py, version 0.11.0 (Vinayak Meta, 2023) in Jupyter Lab, version 4.0.6 (Project Jupyter, 2016) notebooks. In the first step, we created a metadata table of the Red Lists of Berlin to keep track of the extraction process, maintain the source reference links and store summarised data from each Red List pdf file. At the extraction of each file, a data row was added to the metadata table which was updated throughout the rest of the process. In the second step, we identified the page range for extraction for each extracted Red List file.

The extraction mechanism for each Red List file depended on the printed table layout. We extracted tables with lined rows with the Lattice parsing method (Camelot‐py, 2024a), and tables with alternating‐coloured rows with the Stream method (Camelot‐py, 2024b). For proofing the consistency of extraction, we used the Camelot‐py accuracy report along with the Pandas data frame shape property (Pandas, 2024). After initial data cleaning for consistent column counts and missing data, we filtered the data for species in category 0 only. We collated data frames together and exported them as a CSV file. In a further step, we proofread whether the filtered data were tallied with the summary tables, given in each Red List. Finally, we cleaned each Red List table to contain the species, the current hazard level (category 0), the date of the species' last detection in Berlin and the reference (codes and data available at: Github, 2023). When no date of last detection was given for a species, we contacted the authors of the respective Red Lists and/or used former Red Lists to find information on species' last detections (Braasch et al., 2000; Burger et al., 1998; Saure, 2000; Saure et al., 1998, 1999).

### 2.4 Determination of the recording time windows of the Berlin Red Lists

We determined the time windows, the Berlin Red Lists look back on, from their methodologies. If the information was missing in the current Red Lists, we consulted the information from previous versions (Bayer & Winkelmann, 2005; Burmeister, 1838; Deckert & Burghardt, 2018; Degen, 2017; de Sélys‐Longchamps, 1850, 1858; Ehrenberg, 1818; Enderlein, 1906; Erichson, 1837, 1839; Esser, 2017a, 2017b, 2017c, 2017d; Flörke, 1815; Geissler & Kies, 2003; Gottwald, 2017; Hackenberg & Müller, 2017; Heinig & Schöller, 2017; Heinrich & Müller, 2017; Klawitter & Köstler, 2017; Klawitter et al., 2005; Korge, 2003; Korsch & Täuscher, 2016; Kühnel et al., 2017a, 2017b; Machatzi et al., 2005; Mey, 2005; Müller, 2017; Platen & von Broen, 2005; Reineck, 1919; Saure et al., 2005b; Schalow, 1919; Schirmer, 1912; Scholz & Scholz, 2005; Seitz et al., 2012; SenUVK, 2019; Speyer & Speyer, 1862; Stein, 1863; Strübing, 1956; Wanach, 1915; Weidlich, 2022; Wolff, 1998; See all detailed time windows of the earliest assessments in Table B2 in Appendix S2).

### 2.5 Data classification

For the analyses of the percentage of species in the different hazard levels, we used the German Red List categories as described in detail by Saure and Schwarz (2005) and Ludwig et al. (2009). These are: Prewarning list, endangered (category 3), highly endangered (category 2), threatened by extinction or extirpation (category 1) and extinct or extirpated (category 0). To determine the number of indigenous unthreatened species in each Red List, we subtracted the number of species in the five categories and the number of non‐indigenous species (neobiota) from the total number of species in each Red List.

For further analyses, we pooled the taxonomic groups of the 37 Red Lists into more broadly defined taxonomic groups: Plants, lichens, fungi, algae, mammals, birds, amphibians, reptiles, fish and lampreys, molluscs and arthropods (see categorisation in Table 1). We categorised slime fungi (Myxomycetes including Ceratiomyxomycetes) as ‘fungi’, even though they are more closely related to animals, because slime fungi are traditionally studied by mycologists (Schmidt & Täglich, 2023). We classified ‘lichens’ in a separate category, rather than in ‘fungi’, as they are a symbiotic community of fungi and algae (Krause et al., 2017). For analyses of the percentage of extirpated species of each pooled taxonomic group, we set the number of extirpated species in relation to the sum of the number of unthreatened species, species in the prewarning list and species in the categories one to three.

We further categorised the extirpated species according to the habitats in which they occurred. We therefore categorised terrestrial species as ‘terrestrial’ and aquatic species as ‘aquatic’. Amphibians and dragonflies have life stages in both, terrestrial and aquatic habitats, and were categorised as ‘terrestrial/aquatic’. We also categorised plants and mosses as ‘terrestrial/aquatic’ if they depend on wetlands (see all habitat categories for each species in Table C1 in Appendix S3).

The available data considering the species' last detection in Berlin ranked from a specific year, over a period of time up to a century. If a year of last detection was given with the auxiliary ‘around’ or ‘circa’, we used for further analyses the given year for temporal classification. If a year of last detection was given with the auxiliary ‘before’ or ‘after’, we assumed that the nearest year of last detection was given and categorised the species in the respective century. In this case, we used the species for temporal analyses by centuries only, not across years. If only a time frame was given as the date of last detection, we used the respective species for temporal analyses between centuries, only. We further classified all of the extirpated species in centuries, in which species were lastly detected: seventeenth century (1601–1700), eighteenth century (1701–1800), nineteenth century (1801–1900), twentieth century (1901–2000) and twenty‐first century (2001–now) (see all data on species' last detection in Table C1 in Appendix S3).

For analyses of the effects of the number of inhabitants on species' extirpation in Berlin, we used species that went extirpated between the years 1920 and 2012, because of Berlin's was expanded to ‘Groß‐Berlin’ in 1920 (Buesch & Haus, 1987), roughly corresponding to the cities' current area. Therefore, we included the number of Berlin's inhabitants for every year a species was last detected (Statistische Jahrbücher der Stadt Berlin, 1920, 1924–1998, 2000; see all data on the number of inhabitants for each year of species' last detection in Table C1 in Appendix S3).

### 2.6 Statistical analyses

For statistical analyses, we used the R‐project, version 4.3.1 (R Core Team, 2023), and Microsoft Excel 2019, version 1808, for graphical representations. We applied the *G*‐test of independence (R packages ‘RVAideMemoire’; Hervé, 2013) for analyses of the proportions of extirpated species over centuries (seventeenth, eighteenth, nineteenth, twentieth and twenty‐first centuries) in relation to the total number of extirpated species (*N* = 1404, for 29 species no century was available), and the proportions of extirpated species' habitats (terrestrial, terrestrial/aquatic and aquatic) in space by using the total number of extirpated species (*N* = 1433), and over time (*N* = 580). For pairwise comparisons of the proportions of extirpated species between centuries and habitats, we used the pairwise *G*‐test with Bonferroni correction for multiple testing, respectively.

To analyse whether years and/or the number of inhabitants in Berlin (used here as an indicator of urban densification) had an effect on species extirpation, we used the data from 1920 onwards. In the first step, we tested our dependent (number of extirpated species) and independent (year; inhabitants of Berlin) variables for collinearity/multicollinearity using a Spearman correlation matrix. We found no collinearity/multicollinearity between our variables.

In the next step, we tested the distribution of the dependent variable, representing count data, for overdispersion for a Poisson distribution with the link‐function sqr (Zuur et al., 2009). As there was no overdispersion, we applied the generalised linear model (Number of extirpated species ~ Year + Inhabitants of Berlin) for the Poisson family and link‐function sqr (GLM; R package ‘lme4’; Bates et al., 2015) to analyse the effects of years and the inhabitants of Berlin on species extirpation.

## 3 RESULTS

**FIGURE 1:**
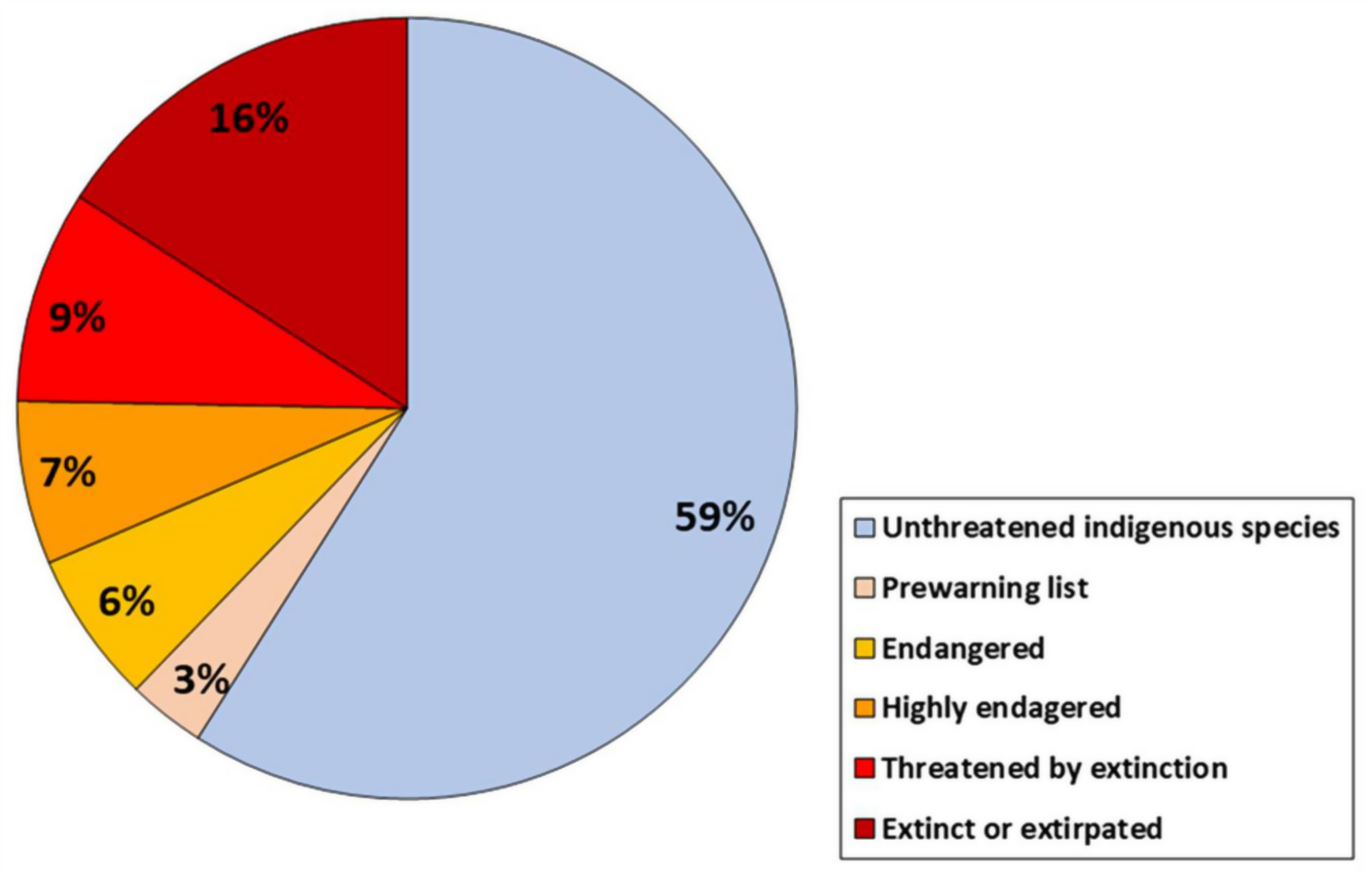
Berlin indigenous species (*N* = 9052) from 37 Red Lists of Threatened Plants, Animals and Fungi in the six Red List categories: Unthreatened indigenous species (blue), species on the prewarning list (flesh colour), endangered species (category 3, yellow), highly endangered species (category 2, orange), species threatened by extinction (category 1, light red) and extinct or extirpated species (category 0, dark red).

Of the total 9498 species included in the 37 Red Lists of Threatened Plants, Animals and Fungi of Berlin, 446 species are non‐indigenous, 9052 are indigenous, of which 59% (5352 species) are unthreatened and 41% are listed in one of the five hazard categories, including 3% on the prewarning list, 6% in category 3 ‘endangered’, 7% in category 2 ‘highly endangered’, 9% in category 1 ‘threatened by extinction’ and 16% (1433 species) in category 0 ‘extinct or extirpated’ (Figure 1). Of the 1433 extirpated species in Berlin, 29 species (2.02%) are also listed as extirpated at the German national scale (Red List of Threatened Plants, Animals and Fungi of Germany; Rote Liste Zentrum, 2024a). None of the extirpated species in Berlin are globally extinct (IUCN, 2024).

**FIGURE 2:**
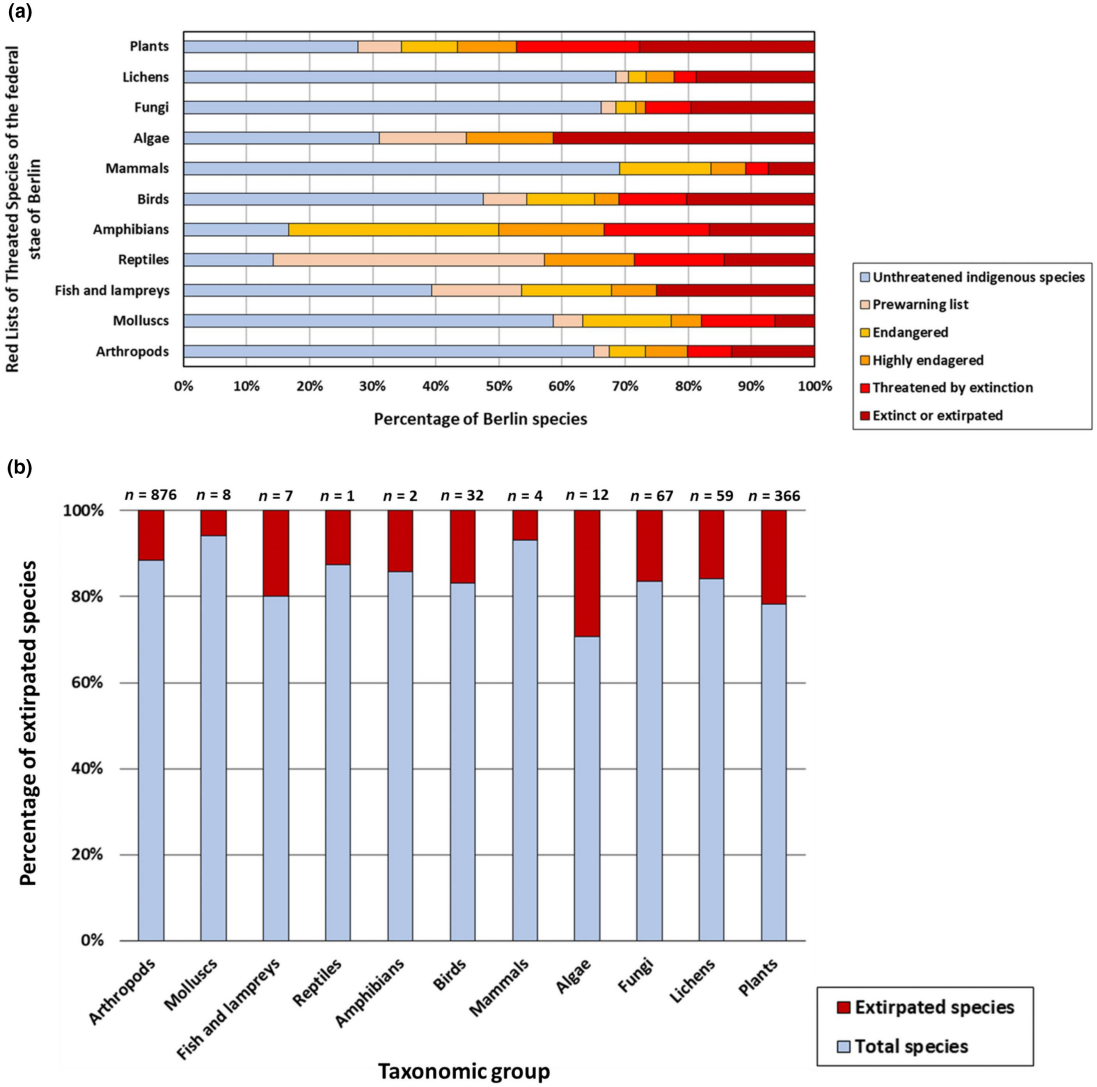
(a) Taxonomic groups of Berlin indigenous species in the six Red List categories: Unthreatened indigenous species (blue), species on the prewarning list (flesh colour), endangered species (category 3, yellow), highly endangered species (category 2, orange), species threatened by extinction (category 1, light red) and extinct or extirpated species (category 0, dark red). Numbers behind bars show the total numbers of indigenous species in the respective taxonomic group. (b) Taxonomic groups of Berlin extirpated species (red), shown as proportion of the total number of species (blue). The numbers above the bars indicate the numbers of extirpated species in the respective taxonomic group.

The percentage of extirpated species in the 37 taxonomic groups of the Red Lists ranged from 54% (smut fungi) and 52% (stonewort algae) over 25% (bagmoths) and 23% (scarab beetles) to 0% (earwings and mayflies) (see Figure A1 in Appendix S1). After pooling the taxonomic groups into plants, lichens, fungi, algae, mammals, birds, amphibians, reptiles, fish and lampreys, molluscs and arthropods, the taxonomic groups of algae (41%) and plants (25%) showed the highest percentage of extirpated species, whereas molluscs (6%) and mammals (7%) showed the lowest percentage of extirpated species (Figure 2a,b).

Our results of the *G*‐test of independence regarding the number of extirpated species per habitat showed the highest significant differences between terrestrial, terrestrial/aquatic and aquatic habitats (*G* = 1367.7, df = 2, *p* < .001). The pairwise *G*‐test with Bonferroni correction for multiple testing further showed that highest significant more species went extirpated in terrestrial habitats (88%; 1253 species) in comparison to terrestrial/aquatic habitats (6%; 91 species; *p* < .001) and in comparison to aquatic habitats (6%; 89 species; *p* < .001). We found no significant differences in the number of extirpated species between terrestrial/aquatic and aquatic habitats.

The time windows the Berlin Red Lists look back on, ranged from the eighteenth over the nineteenth to the twentieth century. The earliest assessments of breeding birds took place at the turn of the nineteenth and twentieth centuries (see all detailed time windows of earliest assessments with references in Table B2 in Appendix S2).

**FIGURE 3:**
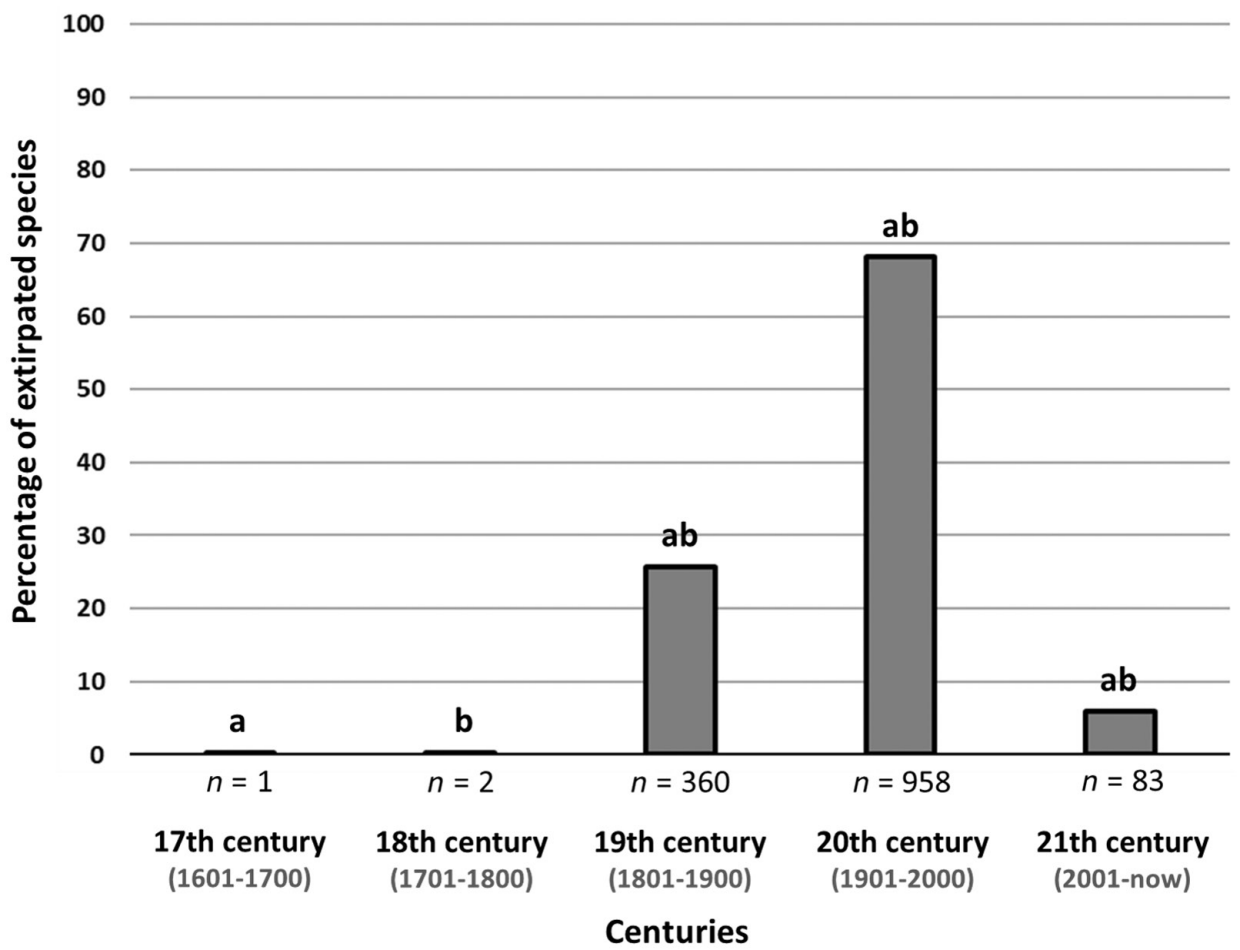
Extirpated species from Berlin Red Lists of Threatened Plants, Animals and Fungi in the seventeenth, eighteenth, nineteenth, twentieth and twenty‐first century. The numbers below bars describe the numbers of extirpated species within each century. The same letters above the bars indicate significant differences in the frequency of extirpated species between centuries (*a* > 0.001; *b* > 0.001).

Most of the species (958 species) in Berlin extirpated across the twentieth century, followed by 360 species across the nineteenth century. One species has been documented to have been extirpated across the seventeenth century, and two across the eighteenth century. In the twenty‐first century, covering 23 years from 2001 to the most recent Red Lists from 2023, 83 species were listed as extirpated (Figure 3). The results of the *G*‐test of independence for the number of extirpated species per century in relation to the total number of extirpated species, for which a century of last detection was available, showed significant differences (*G* = 1929.1, df = 4, *p* < .001). Our results of pairwise *G*‐tests with Bonferroni correction revealed no significant differences between the proportions of extirpated species between the seventeenth and the eighteenth centuries. However, the proportion of extirpated species was significantly higher in the nineteenth, twentieth and twenty‐first centuries compared to the proportion of extirpated species in the seventeenth and eighteenth centuries. Our results further showed that the proportion of extirpated species was significantly higher in the twentieth century in comparison to the nineteenth and twenty‐first centuries. Furthermore, significantly more species went extirpated in the nineteenth century than in the twenty‐first century (see summary statistics in Table B1 in Appendix S2). The average number of extirpated species in the nineteenth century was at 3.6 species per year and in the twentieth century at 9.6 species per year. In the twenty‐first century (2001–2023), 3.6 species went extirpated per year.

**FIGURE 4:**
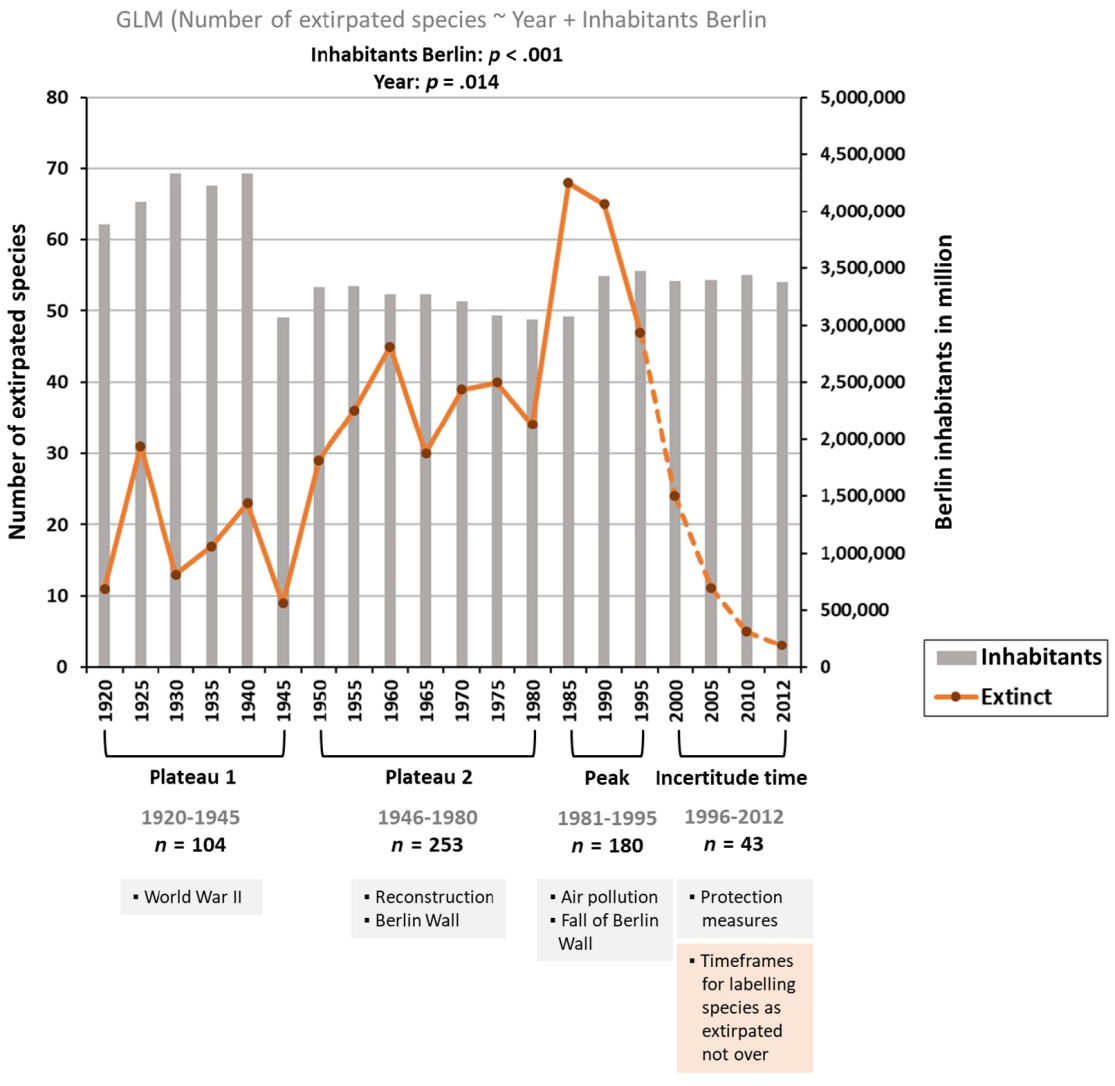
Temporal development of species' extirpation since the extension of the city into ‘Groß‐Berlin’ in 1920. The number of extirpated species in Berlin (orange line) is plotted together with the number of inhabitants in millions (grey bars) in 5‐year intervals, and divided into time periods (1920–1945: First plateau, 1946–1980: Second plateau, 1981–1995: Peak and 1996–2012: Incertitude time). Historical events in Berlin that might have influenced species' extirpation are shown in the grey boxes below each time period. The orange box and the dashed line for the decline in extirpated species of the incertitude time indicate that several species cannot yet be documented as extirpated, because the minimum time frame for labelling species as extirpated might not have elapsed. The numbers below the time periods describe the respective number of extirpated species. Significant *p*‐values are given for generalised linear model results.

Our findings regarding the number of inhabitants of Berlin over the years showed that the number of inhabitants was highest after Berlin's expansion in 1920, ranging between 4 and 4.5 million inhabitants, and a collapse in population after the Second World War in 1945 to about 3 million inhabitants. Berlin's population stayed at this low level until the beginning of the 1990s and slowly increased again (Figure 4).

The number of extirpated species from 1920 to 2012, shown in 5‐year intervals in Figure 4, was marked by two plateaus, a peak and a time period (1996–2012) in which species' extirpation appears to be declining. We have defined the time period from 1996 to 2012 as an ‘incertitude time’ in which several species cannot yet be documented as extirpated (see Section 4). The first plateau was visible from 1920 to 1945 with a total number of 104 extirpated species and a mean of 17 extirpated species in the given 5‐year intervals. The variation ranged from nine extirpated species in 1945 to 31 in 1925. The second plateau was between 1946 and 1980, when 253 species went extirpated with a mean of 36 extirpated species in the given 5‐year intervals. The variation ranged from 29 extirpated species in 1950 to 45 in 1960. There was a peak between 1985 and 1995, when 180 species went extirpated with an average of 60 extirpated species in the given 5‐year intervals. The variation ranged from 47 extirpated species in 1995 to 68 in 1985. In the 5‐year intervals between the years 1996 and 2012, an incertitude time was visible in which 43 species went extirpated, with a mean of 11 extirpated species in the given 5‐year intervals. The variation ranged from three extirpated species in 2012 to 24 in 2000 (Figure 4).

**FIGURE 5:**
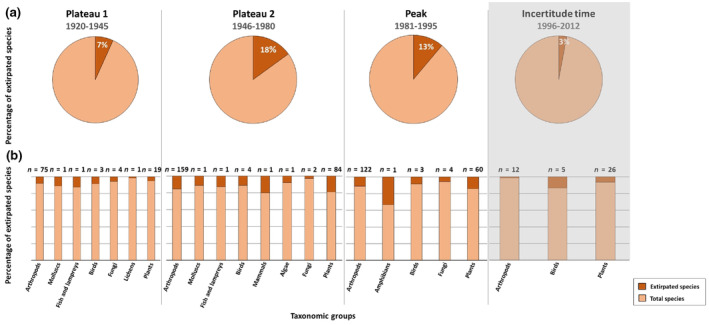
Species' extirpated since the city's extension of the city into ‘Groß‐Berlin’ in 1920 in time periods, taken from Figure 4 (1920–1945: First plateau, 1946–1980: Second plateau, 1981–1995: Peak and 1996–2012: Incertitude time) in relation to (a) the total number of extirpated species in Berlin (*N* = 1433), and (b) the total number of extirpated species in each taxonomic group (Arthropods: *n* = 875, Molluscs: *n* = 8, Fish and lampreys: *n* = 7, Birds: *n* = 32, Fungi: *n* = 67, Lichens: *n* = 59, Plants: *n* = 366, Mammals: *n* = 4, Algae: *n* = 12 and Amphibians: *n* = 2). The grey background behind the incertitude time indicates that several species cannot yet be documented as extirpated because the minimum time frame for labelling species as extirpated might not have elapsed. The numbers above the bars indicate the number of extirpated species in each taxonomic group.

The results of the GLM (Number of extirpated species ~ Year + Inhabitants of Berlin) showed that the number of inhabitants of Berlin had a highly negative effect on the number of extirpated species and that the factor ‘Year’ had a significantly negative effect on the number of extirpated species (see Table 2, Figure 4). When considering the number of extirpated species in each of the time periods (first plateau, second plateau, the peak and the incertitude time) in relation to the total number of extirpated species from all the 37 Red Lists (*N* = 1433), our results showed that 7% of the species went extirpated in the first plateau (1920–1945), covering seven taxonomic groups. The percentage of extirpated species increased in the second plateau (1946–1980) to 18%, covering eight taxonomic groups. Although the time period of the peak (1981–1995) is shorter, 13% went extirpated in this time frame, covering five taxonomic groups. In the following incertitude time (1996–2012), only 3% of the total extirpated species disappeared, covering three taxonomic groups (Figure 5a,b).

**TABLE 2:** Summary statistics of generalised linear model with Poisson family, link‐function sqr, testing the effects of the number of inhabitants in Berlin and the year on the number of extirpated species from 1920 to 2012.

Dependent variable	Independent variable	SE	*z*‐Value	*p*‐Value
Number of extirpated species	Number of inhabitants in Berlin	<.001	−6.775	<.001
	Year	<.001	−2.458	.014

**FIGURE 6:**
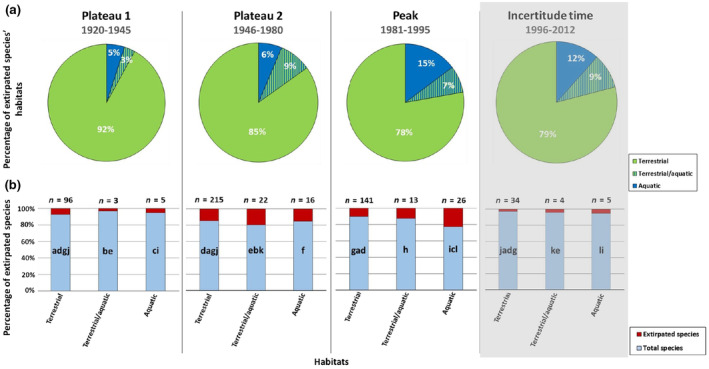
Habitats of extirpated species since the expansion of the city into ‘Groß‐Berlin’ in 1920 in time periods, taken from Figure 4 (1920–1945: First plateau, 1946–1980: Second plateau, 1981–1995: Peak and 1996–2012: Incertitude time) (a) Percentage of habitats of extirpated species in relation to (b) the total numbers of habitats of extirpated species (terrestrial: *n* = 1253, terrestrial/aquatic: *n* = 91 and aquatic: *n* = 89). The grey background behind the incertitude time indicates that several species cannot yet be documented as extirpated because the minimum time frame for labelling species as extirpated might not have elapsed. The numbers above the bars indicate the number of extirpated species in each habitat. The same letters within the bars indicate significant differences in the frequency of extirpated species between centuries.

In the four time periods given (plateau 1, plateau 2, peak and incertitude time), most of the species disappeared from terrestrial habitats, with a decreasing percentages over time (Figure 6a). The loss of terrestrial species was followed by an increasing loss of species in aquatic habitats, and an increasing loss of species dependent on both, terrestrial and aquatic habitats.

In relation to the total number of extirpated species that occurred in terrestrial (*n* = 1232), terrestrial/aquatic (*n* = 91) and aquatic (*n* = 89) habitats in Berlin, the proportions of extirpated species in terrestrial habitats between the four time periods showed 7% in the first plateau, increased to 17% in the second plateau, and decreased to 11% at the peak, and 3% during the incertitude time (Figure 6b). The results of the *G*‐test of independence for the number of extirpated species in terrestrial habitats in the four time periods showed significant differences (*G* = 142.89, df = 3, *p* < .001), and the results of the pairwise *G*‐tests with Bonferroni correction revealed significant differences between the proportions of extirpated species in terrestrial habitats between the four time periods (Table 3).

**TABLE 3:** Summary statistics of pairwise *G*‐test with Bonferroni correction for multiple testing for the number of extirpated species in terrestrial, terrestrial and aquatic and aquatic habitats between the time periods 1920–1945, 1946–1980, 1920–1945 and 1996–2012.

Habitat	Group of comparison		*p*‐Value
Terrestrial	1920–1945	1946–1980	<.001
	1946–1980	1981–1995	.001
	1981–1995	1996–2012	.001
	1920–1945	1981–1995	.030
	1920–1945	1996–2012	<.001
	1946–1980	1996–2012	<.001
Terrestrial/aquatic	1920–1945	1946–1980	.001
	1946–1980	1981–1995	.965
	1981–1995	1996–2012	.191
	1920–1945	1981–1995	.075
	1920–1945	1996–2012	1
	1946–1980	1996–2012	.003
Aquatic	1920–1945	1946–1980	.117
	1946–1980	1981–1995	.976
	1981–1995	1996–2012	.002
	1920–1945	1981–1995	.002
	1920–1945	1996–2012	1
	1946–1980	1996–2012	.117

Our results of the *G*‐test of independence for the proportions of extirpated species between the four time periods in terrestrial/aquatic habitats, showed highest significant differences (*G* = 20.55, df = 3, *p* < .001). The proportions of extirpated species in terrestrial/aquatic habitats were 3% in the first plateau, increased to 9% in the second plateau, decreased to 7% at the peak and increased again to 9% during the incertitude time (Figure 6b). The results of pairwise *G*‐tests with Bonferroni correction revealed significant differences between the proportions of extirpated species between plateau 1 and plateau 2, between plateau 2 and the incertitude time and between plateau 2 and the incertitude time. We found no significant differences in the proportions of extirpated species of terrestrial/aquatic habitats between the peak and the other three time periods, and between plateau 1 and the incertitude time (Table 3).

The results of the *G*‐test of independence for the proportions of extirpated species that occurred in aquatic habitats between the three time periods showed significant differences (*G* = 20.61, df = 3, *p* < .001). The proportions of extirpated species in aquatic habitats were 5% in the first plateau, increased to 6% in the second plateau and further increased to 15% at the peak, and 12% during the incertitude time (Figure 6b). The results of pairwise *G*‐tests with Bonferroni correction revealed significant differences in the proportions of extirpated species between the peak, plateau 1 and the incertitude time. We found no significant differences between plateau 1, plateau 2 and the incertitude time, between plateau 2, the peak and the incertitude time (Table 3).

Our results showed that the amount of species' extirpation in Berlin was, as expected, at a high level. The process of species' extirpation increased over centuries and was negatively affected by the number of inhabitants in the city, while different taxonomic groups and different habitats were affected differently.

## 4 DISCUSSION

Urbanisation is steadily increasing globally and is one of the major man‐made interventions in the environment (Goddard et al., 2010; Liu et al., 2016; Soundranayagam et al., 2011), with multifaceted consequences for urban biodiversity (Aronson et al., 2017; McDonald et al., 2008; McKinney, 2002, 2008; Seto et al., 2012; Theodorous, 2022). In our study, we made spatio‐temporal species' extirpation at multitaxonomic level visible at the urban scale.

We expected a very high level of species' extirpation in Berlin, as the area is relatively small (892 km^2^) and highly urbanised. As expected, we found a high level of species' extirpation at 16% (1433 species). We also found that 446 non‐indigenous species newly invaded to Berlin, resulting in a deficit of 987 species. While there is a net loss of species in Berlin, it remains to be investigated whether some non‐indigenous species might functionally replace some extirpated species, or whether non‐indigenous species ‘only’ contribute to the formation of novel ecosystems.

Species' extirpation is known to be higher at small scales than on larger scales (Halley et al., 2013), as extinction is a process that starts at the local scale (Purvis et al., 2000; Sodhi et al., 2009). While the percentage of extirpated species in Europe is reported to be 0.34% (Hochkirch et al., 2023), in Germany, it is 2.7%, that is, eight times higher. At the urban scale of Berlin, the percentage is even increased 47.1 times higher than at the European scale and 5.9 times higher than at the German scale. It must therefore be clearly emphasised that the taxa groups assessed for these Red Lists are by no means harmonised between the different spatial scales (Hochkirch et al., 2023). The percentage of extirpation (16%; 1433 species) in Berlin is comparable to the percentage of species' extirpation (15%; 1273 species) in the German city‐state of Hamburg (Behörde für Umwelt, Klima, Energie und Agrarwirtschaft, Hamburg, 2024), the only data we could find analysing a similarly broad range of taxonomic groups at the city scale. The metropolis of Hamburg is the second largest city in Germany after Berlin, both in terms of area (755 km^2^) and number of inhabitants (1,899,160) (Hamburg in Zahlen, 2024). At 44.9%, Hamburg also has a similar average degree of sealed surfaces to Berlin at 46.9% (used here as an additional indicator for the level of urbanisation) (Statista, 2024d).

The high similarity of extirpation levels in both, Berlin and Hamburg might indicate a similar pattern, that might be generalised to other urbanised areas, at least in Central Europe, and might also apply to other areas. However, the question of whether this high level of species' extirpation is an urban effect or solely dependent on the size of the study area is much more difficult to investigate. This is particularly the case as extirpation levels outside urban areas with less human impact are so far very poorly studied. Multitaxonomic data on species' extirpation from regional Red Lists covering rural areas are available for two other German federal states: Saarland, covering an area of 2570 km^2^, of which 8.03% are sealed surfaces and over 50% are agricultural landscapes. Saarland has 386 inhabitants per km^2^ (Saarland, 2022; Statistische Ämter, 2024). Here, species' extirpation amounts to only 6.2% (Rote Liste Saarland, 2024). In Niedersachsen, the proportion of extirpated species is at a similar, slightly lower level of 5.8% (NLWKN, 2024), although Niedersachsen (47,614 km^2^; 171 inhabitants per km^2^) is 18.5 times larger than Saarland, and only 6.52% of the area is sealed (Niedersachsen, 2024; Statistische Ämter, 2024). Other studies focussing on scale and landscape‐dependent extirpation events in individual taxonomic groups show a similar pattern, with the proportion of extirpated vascular plant species decreasing with the size of the study area (Colling, 2005), and extirpation rates in grassland plants increasing along rural–urban gradients (Williams et al., 2005).

The total number of species occurring in Berlin remains unstudied, as the Berlin Red Lists cover 9498 species, whereas the Red List of Germany covers 20,000 species (Rote Liste Zentrum, 2024a). Many, even large, taxonomic groups, especially from fungi, soil biodiversity, plankton and small hymenopteran and dipteran insects, are unstudied or only partially recorded for the Berlin Red Lists. It must therefore be taken into account that the 16% of species' extirpation represents only the detected, visible part, while many more species might went extirpated without ever having been recorded.

On a temporal scale, we predicted species' extirpation in the city to be highest during periods when human interventions in the environment were at their highest (Czech et al., 2000; Fenger, 1999; reviewed in Doherty et al., 2016; Falchi, 2019; Goddard et al., 2010; Liu et al., 2016; Marzluff, 2001; Pyšek et al., 2020; Rizwan et al., 2008). This prediction seems to be confirmed for Berlin in our study. However, the documentation of such early biodiversity levels remains incomplete and we cannot exclude that many more species were already extirpated during these times. While species' extirpation was low in the seventeenth and eighteenth centuries, it increased significantly from the nineteenth century onwards, reaching its highest value in the twentieth century. The nineteenth century was characterised by a rapidly increase in the number of urban dwellers (Ribbe et al., 2002a; Ring, 1992) and major interventions in the environment during the industrialisation, with the rapid and large‐scale construction of industrial and residential facilities (Ribbe et al., 2002a, 2002b; Wey, 1982). This process might have resulted in high levels of habitat destruction and fragmentation, and thus species' extirpation. The resulting pollution, including exhaust fumes (Pamme, 2003; Wey, 1982), might have exacerbated this process. In addition, the development of the Haber–Bosch method in the early twentieth century (Erisman et al., 2008; Ramankutty et al., 2018) led to heavy use of chemical fertilisers in the city's surrounding, which may also have affected the urban area (Le, 2008) and biodiversity. The twentieth century was a period of drastic changes in the urban environment. After the expansion of Berlin to ‘Groß‐Berlin’ in 1920, urban development took the next major step, with large‐scale construction and further increases in the human population, until the beginning of the Second World War and its drastic destruction and subsequent reconstruction (Buesch & Haus, 1987; Statistische Jahrbücher der Stadt Berlin, 1920, 1924–1998, 2000). We also showed that the number of inhabitants had a negative effect on species' extirpation in Berlin, probably as a result of increased urban densification.

As we used the average degree of sealed surfaces as an additional indicator for the level of urbanisation for the scale‐dependent comparison and discussion of species' extirpation above, we were not able to include this indicator in the analyses and comparisons of species' extirpation between the four time periods given here. Because of the division of Berlin between 1961 and 1989, data on the degree of sealed surfaces (Umweltatlas Berlin, 2021) and data on other potentially relevant indicators such as open spaces, residential and transportation areas have only been collected using standardised methods for the whole of Berlin since the 1990s (Ribbe et al., 2002b; Schildt & Sywottek, 1993; Umweltatlas Berlin, 2020).

From 1920 onwards, the course of species' extirpation was characterised by two plateaus (1920–1945 and 1946–1980), a peak (1981–1995) and an incertitude time (1996–2012). In order to find explanations for the temporal development of species' extirpation in the city, the historical circumstances of Berlin had to be considered. During the first plateau, the Second World War took place and Berlin was affected by considerable destruction and treatment due to bombing, resulting in a drastic decline in human population during and after the war (Ribbe et al., 2002b; Schildt & Sywottek, 1993). Although species' extirpation is shown to be at a low level during the first plateau, it would have been expected that the enormous influence of the war might have led to an increase in species' extirpation, as the negative effects on species have been described for destroyed, fragmented and lost habitats (Czech et al., 2000; Goddard et al., 2010; Liu et al., 2016; Marzluff, 2001). The explanation for this low level of species' extirpation during the first plateau might be that species recording was a low priority during this time. However, the impact of this destruction on Berlin's biodiversity has been described for the stock of street trees, which declined massively due to the war from around 411,000 trees in 1939 to around 161,000 trees in 1946. Berlin was able to continuously rebuild its tree stock and with reunification, the city had around 370,000 street trees at the end of 1990 (SenMVKU, 2014c). Species' extirpation in Berlin increased from a total of 7% (plateau 1) to 18% (plateau 2), affecting seven taxonomic groups in the first plateau and eight taxonomic groups in the second plateau. This increase in species' extirpation on the second plateau might be explained by the fact that the effects of the wartime destruction on species in the city only became apparent in the post‐war period and might have been exacerbated by the reconstruction of the city (plateau 2), which in turn led to massive interventions in the environment (Ribbe et al., 2002b). Although our results showed that the number of inhabitants had a significant negative effect on species' extirpation, the decline in Berlin's human population during the Second World War might have played a subordinate role in species' extirpation, as the massive destruction of the war might have dominated the situation.

Although the following time frame covered only 15 years, a clear peak in species' extirpation became apparent, which 13% of the total extirpated species disappearing from Berlin, covering five taxonomic groups. The peak covers the years from 1981 to 1995, a time frame characterised by high levels of air pollution throughout Germany, resulting in acid precipitation (Rissberger, 1985) and negative effects on terrestrial and aquatic ecosystems and species (Singh & Agrawal, 2008). This pollution might have also affected Berlin, leading to the resulting peak in species' extirpation. In addition, this time frame covered a period of social change, the fall of the Berlin Wall, and the reunification of East and West Berlin (Ribbe et al., 2002b). This major upheaval, combined with the further expansion within the city (Ellger, 1992), might have contributed to the extirpation of species with previously unstable populations.

We defined the time period (1996–2012) immediately after the peak, as an ‘incertitude time’, because the minimum time frames after which species are considered extirpated, that is, 10 or 20 years (Binot et al., 1998; Prasse et al., 2001), might not be met for several species. Therefore, a decline in species' extirpation was observed. The incertitude time began at the turn of the millennium and lasted until 2012, when only 3% of the total number of extirpated species in Berlin were described as extirpated. Within this ‘incertitude time’, extirpated species covered the three taxonomic groups of arthropods, birds and plants. An additional explanation for this decrease in the incertitude time might be a more cautious approach to labelling species as extirpated, as species that are extirpated in Berlin might still occur on the edge of the federal state of Brandenburg, which completely surrounds Berlin. There is therefore a high probability that such species could be resettled to the city. Further explanations for the decrease might be that the populations of species might be so small that no individuals can be found despite intensive searches and the increasing importance of environmental and species protection measures in Berlin during this time period, with positive effects on indigenous species (SenMVKU, 2024b).

By 2023, the year of the latest Berlin Red List used in our study, 83 species are considered as extirpated in the twenty‐first century. This corresponds to an annual average of 3.6 extirpated species, as in the nineteenth century, despite the much shorter time frame. In the twentieth century, however, the annual average of extirpated species is 9.6, the highest number ever recorded in Berlin. As the Berlin Red Lists consider different time windows, it is important to note that species' extirpation prior to these time windows is not considered in this study due to a lack of reliable data.

We are aware that biodiversity in Berlin has changed over time and has deviated greatly from its natural state. However, it is not possible to trace the change in biodiversity from the first settlements in the area of present‐day Berlin in the thirteenth century (Ribbe et al., 2002a; Ring, 1992) to the earliest time window in which Red Lists look back, that is, 1750. However, we assume that species' extirpation was already at a high level in previous centuries. For example, the Red List of mammals looks back to the nineteenth century but does not consider species extirpated before 1927, whereas European bison, wild horses, aurochs and the brown bear, which certainly occurred in the Berlin area, are included (Klawitter et al., 2005).

We also found that most of the species in Berlin went extirpated from terrestrial habitats (88%). Terrestrial habitats might be most affected by species' extirpation because Berlin consists of 94% of the terrestrial area and only a small part (3.1%) of the total urban area are protected natural areas (SenStadt, 2020) that are not under continuous construction. On a temporal scale, however, the extirpation of terrestrial species remained more or less constant when considering the two plateaus, the peak and the incertitude time. However, when looking at species' extirpation in terrestrial habitats between the time periods (plateaus, peak and incertitude time) in relation to the total number of species extirpated in terrestrial habitats in each time period, there was an increase in species' extirpation. Thus, our results showed that terrestrial habitats are the most threatened and continuously threatened habitat types in the city.

Total species' extirpation in both aquatic and terrestrial/aquatic habitats are 6%, much lower than in terrestrial habitats. However, on a temporal scale, the percentage of species' extirpation in aquatic and terrestrial/aquatic habitats increased over time. Although most species went extirpated in terrestrial habitats, our results show that the increase in the number of extirpated species over time was highest in aquatic habitats. Berlin consists of 6% of blue spaces (SenStadt, 2020). In particular, lakes and small water bodies, such as urban ponds, might be affected by increasing temperatures due to climate change, which is exacerbated by the urban environment with sealed surfaces and buildings (Dabrowska et al., 2023; McKinney, 2002), resulting in increasing water temperatures, threatening the indigenous aquatic and terrestrial/aquatic species living in them, up to the complete drying out of water bodies and thus the extirpation of species (Dabrowska et al., 2023; McKinney, 2002). These findings might be confirmed by our further results, which examined whether taxonomic groups are differently affected by species' extirpation. These results showed that especially algae, fish and lampreys show high levels of species' extirpation. This confirms our prediction that species' extirpation is dependent on taxonomic groups and might be an additional hint that aquatic systems in Berlin may be particularly threatened over time.

In conclusion, our results showed that the number of extirpated species is higher at small scales than at large scales, and might additionally be negatively influenced by urbanisation. Therefore, cities might be suitable systems for studying species' extirpation processes due to their small scales and human influence. We further showed that species' extirpation was dependent on the taxonomic group and the habitats in which the species occurred. On a temporal scale, our results showed that most species were extirpated during time periods of high human impact, and that the recent century, despite its relatively short time frame, is already comparable to the extent of species' extirpation in the whole nineteenth century. Species' extirpation is also influenced by the number of inhabitants in the city, and might also be reflected by natural and political contexts and happenings in time.

As our analyses are based on the Red Lists of Threatened Plants, Animals, and Fungi, we are aware that Red Lists are mostly based on the experience of a few experts, mostly citizen scientists. The quality of knowledge of professional and citizen scientists might vary, as might the number of experts for each taxonomic group over time. Therefore, it cannot be excluded that there is a spatio‐temporal bias in the recording intensity. Furthermore, detection of extirpation is challenging, as experts need to be sure, that a species has not been overseen, or might be considered extirpated simply because no one has searched for it. However, the study by Sommerwerk et al. (2021) showed that associations and their specialist groups, which are often responsible for the Red List data, are usually organised in differentiated structures and have a high level of expertise. Although the knowledge contained in Red Lists might sometimes be suspected of being inadequate, it is the best data we have for many taxonomic groups. Therefore, the data and numbers presented here might not be absolutely accurate in every detail, but the general situation is expected to remain unchanged. Regional Red Lists might therefore be used as a basis for tracking the development of local biodiversity, such as in a biodiversity index.

## AUTHOR CONTRIBUTIONS

**Silvia Keinath:** Conceptualization (equal); formal analysis (equal); funding acquisition (equal); methodology (equal); visualization (equal); writing – original draft (equal); writing – review and editing (equal). **Shenya De Silva:** Data curation (equal); formal analysis (equal); software (equal); writing – review and editing (equal). **Nike Sommerwerk:** Funding acquisition (equal); writing – review and editing (equal). **Jörg Freyhof:** Conceptualization (equal); funding acquisition (equal); methodology (equal); writing – original draft (equal); writing – review and editing (equal).

## FUNDING INFORMATION

This project was funded by the innovation fond of the Museum für Naturkunde, Berlin–Leibniz Institute for Evolution and Biodiversity Science.

## CONFLICT OF INTEREST STATEMENT

We declare no conflicts of interest.

## Supporting information


Appendix S1

Appendix S2

Appendix S3

## Data Availability

All data sets generated for this study are included in the Supporting Information of the article. Codes and data on extracted extirpated species from the Berlin Red Lists are stored under: https://github.com/Zzzhenya/Berlin‐red‐list.

## References

[ece370018-bib-0001] Aronson, M. F. J. , Lepczyk, C. A. , Evans, K. L. , Goddard, M. A. , Lerman, S. B. , Macivor, J. S. , Nilon, C. H. , & Vargo, T. (2017). Biodiversity in the city: Key challenges for urban green space management. Frontiers in Ecology and the Environment, 15, 189–196. 10.1002/fee.1480

[ece370018-bib-0002] Auhagen, A. (1991). Rote Listen der gefährdeten Pflanzen und Tiere in Berlin – Zusammenfassung und Empfehlungen. In A. Auhagen , R. Platen , & H. Sukopp (Eds.), Rote Listen der gefährdeten Pflanzen und Tiere in Berlin. Landschaftsentwicklung und Umweltforschung(Vol. 6, pp. 5–11). Technische Universität Berlin.

[ece370018-bib-0003] Bates, D. , Mächler, M. , Bolker, B. , & Walker, S. (2015). Fitting linear mixed‐effects models using lme4. Journal of Statistical Software, 67, 1–48. 10.18637/jss.v067.i01

[ece370018-bib-0004] Bayer, C. , & Winkelmann, H. (2005). Rote Liste und Gesamtartenliste der Rüsselkäfer (Curculionoidea) von Berlin. In Der Landesbeauftragte für Naturschutz und Landschaftspflege/Senatsverwaltung für Stadtentwicklung (Ed.), Rote Listen der gefährdeten Pflanzen und Tiere von Berlin. Der Landesbeauftragte für Naturschutz und Landschaftspflege. Technische Universität Berlin.

[ece370018-bib-0005] Behörde für Umwelt, Klima, Energie und Agrarwirtschaft, Hamburg . (2024). Artenschutz – Rote Listen Hamburgs . https://www.hamburg.de/rote‐liste/

[ece370018-bib-0006] Bertin, R. I. (2002). Losses of native plant species from Worcester, Massachusetts. Rhodora, 104, 920.

[ece370018-bib-0007] Binot, M. , Bless, R. , Boye, P. , Gruttke, H. , & Pretscher, P. (1998). Grundlagen und Bilanzen zur Roten Liste gefährdeter Tiere Deutschlands. Schriftenreihe für Landschaftspflege und Naturschutz, 55, 9–32.

[ece370018-bib-0008] Bonebrake, T. C. , & Cooper, D. S. (2014). A Hollywood drama of butterfly extirpation and persistence over a century of urbanization. Journal of Insect Conservation, 18, 683–692. 10.1007/s10841-014-9675-z

[ece370018-bib-0009] Braasch, D. , Hendrich, M. , & Balke, M. (2000). Rote Liste und Artenliste der Wasserkäfer des Landes Brandenburg (Coleoptera: Hydradephaga, Hydrophiloidea part., Dryopoidea part. und Hydraenidae). Landesumweltamt Brandenburg – Naturschutz und Landschaftspflege, 9, 3.

[ece370018-bib-0010] Buesch, O. , & Haus, W. (1987). Berlin als Hauptstadt der Weimarer Republik 1919–1933. Walter de Gruyter and Co.

[ece370018-bib-0011] Bund‐Länder Demografie Portal . (2024). Bevölkerungszahl in Berlin . https://www.demografie‐portal.de/DE/Fakten/bevoelkerungszahl‐berlin.html

[ece370018-bib-0012] Burger, F. , Saure, C. , & Oehlke, J. (1998). Rote Liste und Artenliste der Grabwespen und weiterer Hautflüglergruppen des Landes Brandenburg (Hymenoptera: Sphecidae, Vespoidae part., Evanioidae, Trigonalyoidae). Landesumweltamt Brandenburg – Naturschutz und Landschaftspflege in Brandenburg, 7, 2.

[ece370018-bib-0013] Burmeister, H. (1838). Handbuch der Entomologie. Enslin.

[ece370018-bib-0014] Camelot‐py . (2024a). Stream type: Camelot‐py parsing method: Lattice . https://camelot‐py.readthedocs.io/en/master/user/how‐it‐works.html#lattice

[ece370018-bib-0015] Camelot‐py . (2024b). Stream type: Camelot‐py parsing method: Stream . https://camelot‐py.readthedocs.io/en/master/user/how‐it‐works.html#stream

[ece370018-bib-0016] Chapin, S. F. , Zavaleta, S. E. , Eviners, T. V. , Naylor, L. R. , Vitousek, M. P. , Reynolds, L. H. , Hopper, U. D. , Lavorel, S. , Sala, E. O. , Hobbie, S. E. , Mack, C. M. , & Díaz, S. (2000). Consequences of changing biodiversity. Nature, 405, 234–242. 10.1038/35012241 10821284

[ece370018-bib-0017] Colling, G. (2005). Red list of the vascular plants of Luxembourg. Musée national d'histoire naturelle.

[ece370018-bib-0018] Cooke, R. , Sayol, F. , Andermann, T. , Blackburn, T. M. , Steinbauer, M. J. , Antonelli, A. , & Faurby, S. (2023). Undiscovered bird extinctions obscure the true magnitude of human‐driven extinction waves. Nature Communications, 14, 8116. 10.1038/s41467-023-43445-2 PMC10730700 38114469

[ece370018-bib-0019] Czech, B. , Krausman, P. R. , & Devers, P. K. (2000). Economic associations among causes of species endangerment in the United States. Bioscience, 50, 593–601. 10.1641/0006-3568(2000)050[0593:EAACOS]2.0.CO;2

[ece370018-bib-0020] Dabrowska, J. , Orellana, A. E. M. , Kilian, W. , Moryl, A. , Cielecka, N. , Michałowska, K. , Policht‐Latawiec, A. , Michalski, A. , Bednarek, A. , & Wlóka, A. (2023). Between flood and drought: How cities are facing water surplus and scarcity. Journal of Environmental Management, 345, 118557. 10.1016/j.jenvman.2023.118557 37429091

[ece370018-bib-0021] de Sélys‐Longchamps, E. (avec collaboration de Hagen H.A.). (1850). Revue des Odonates ou Libellules d'Europe. Mémoires de la Société Royale des Sciences de Liège, 6, 408.

[ece370018-bib-0022] de Sélys‐Longchamps, E. (avec collaboration de Hagen H.A.). (1858). Monographiè des Gomphines. Mémoires de la Société Royale des Sciences de Liège, 11, 257–720.

[ece370018-bib-0023] Deckert, J. , & Burghardt, G. (2018). Rote Liste und Gesamtartenliste der Wanzen (Heteroptera) von Berlin. In Der Landesbeauftragte für Naturschutz und Landschaftspflege/Senatsverwaltung für Umwelt, Verkehr und Klimaschutz (Ed.), Rote Listen der gefährdeten Pflanzen, Pilze und Tiere von Berlin (43 p). Technische Universität Berlin. 10.14279/depositonce-6690

[ece370018-bib-0024] Degen, G. (2017). Rote Liste und Gesamtartenliste der Raubfliegen (Diptera: Asilidae) von Berlin. In Der Landesbeauftragte für Naturschutz und Landschaftspflege/Senatsverwaltung für Umwelt, Verkehr und Klimaschutz (Ed.), Rote Listen der gefährdeten Pflanzen, Pilze und Tiere von Berlin. Technische Universität Berlin. 10.14279/depositonce-5858

[ece370018-bib-0025] Doherty, T. S. , Glen, A. S. , Nimmo, D. G. , Ritchie, E. G. , & Dickman, C. R. (2016). Invasive predators and global biodiversity loss. Proceedings of the National Academy of Sciences of the United States of America, 113, 11261–11265. 10.1073/pnas.1602480113 27638204 PMC5056110

[ece370018-bib-0026] Doudna, J. W. , & Danielson, J. B. (2015). Rapid morphological change in the masticatory structures of an important ecosystem service provider. PLoS One, 10, e0127218. 10.1371/journal.pone.0127218 26061880 PMC4465031

[ece370018-bib-0027] Dri, G. F. , Fontana, C. S. , & de Sales Dambros, C. (2021). Estimating the impacts of habitat loss induced by urbanization on bird local extinctions. Biological Conservation, 256, 109064. 10.1016/j.biocon.2021.109064

[ece370018-bib-0028] Duncan, R. P. , Clemants, S. E. , Corlett, R. T. , Hahs, A. K. , McCarthy, M. A. , McDonnell, M. J. , Schwartz, M. W. , Thompson, K. , Vesk, P. A. , & Williams, N. S. (2011). Plant traits and extinction in urban areas: A meta‐analysis of 11 cities. Global Ecology and Biogeography, 20, 509–519. 10.1111/j.1466-8238.2010.00633.x

[ece370018-bib-0029] Ehrenberg, C. (1818). Silvae mycologicae Berolinensis . Berlin (Theophilus Bruschke).

[ece370018-bib-0030] Ellger, C. (1992). Berlin: Legacies of division and problems of unification. The Geographical Journal, 158, 40–46. 10.2307/3060015

[ece370018-bib-0031] Enderlein, G. (1906). Monographie der Coniopterygiden. Zoologische Jahrbücher, Abteilung für Systematik, Geographie und Biologie der Tiere, 23, 173–242.

[ece370018-bib-0032] Erichson, F. W. (1837). Die Käfer der Mark Brandenburg (Vol. 1, 384 p).

[ece370018-bib-0033] Erichson, F. W. (1839). Die Käfer der Mark Brandenburg (Vol. 1, 356 p).

[ece370018-bib-0034] Erisman, J. W. , Sutton, A. M. , Galloway, J. , Klimont, Z. , & Winiwarter, W. (2008). How a century of ammonia synthesis changed the world. Nature Geoscience, 1, 636–639. 10.1038/ngeo325

[ece370018-bib-0035] Esser, J. (2017a). Rote Liste und Gesamtartenliste der Blatthornkäfer (Coleoptera: Scarabaeoidea) von Berlin. In Der Landesbeauftragte für Naturschutz und Landschaftspflege/Senatsverwaltung für Umwelt, Verkehr und Klimaschutz (Ed.), Rote Listen der gefährdeten Pflanzen, Pilze und Tiere von Berlin. Technische Universität Berlin. 10.14279/depositonce-5792

[ece370018-bib-0036] Esser, J. (2017b). Rote Liste und Gesamtartenliste der Kurzflügelkäferartigen und Stutzkäfer (Coleoptera: Staphylinoidea und Histeridae) von Berlin. In Der Landesbeauftragte für Naturschutz und Landschaftspflege/Senatsverwaltung für Umwelt, Verkehr und Klimaschutz (Ed.), Rote Listen der gefährdeten Pflanzen, Pilze und Tiere von Berlin. Technische Universität Berlin. 10.14279/depositonce-5852

[ece370018-bib-0037] Esser, J. (2017c). Rote Liste und Gesamtartenliste der Kapuzinerkäferartigen (Bostrichoidea), Buntkäferartigen (Cleroidea), Plattkäferartigen (Cucujoidea), Schnellkäferartigen (Elateroidea), Werftkäferartigen (Lymexyloidea) und Schwarzkäferartigen (Tenebrioidea) von Berlin (Coleoptera). In Der Landesbeauftragte für Naturschutz und Landschaftspflege/Senatsverwaltung für Umwelt, Verkehr und Klimaschutz (Ed.), Rote Listen der gefährdeten Pflanzen, Pilze und Tiere von Berlin. Technische Universität Berlin. 10.14279/depositonce-5853

[ece370018-bib-0038] Esser, J. (2017d). Rote Liste und Gesamtartenliste der Bockkäfer (Coleoptera: Cerambycidae) von Berlin. In Der Landesbeauftragte für Naturschutz und Landschaftspflege/Senatsverwaltung für Umwelt, Verkehr und Klimaschutz (Ed.), Rote Listen der gefährdeten Pflanzen, Pilze und Tiere von Berlin. Technische Universität Berlin. 10.14279/depositonce-5856

[ece370018-bib-0039] Falchi, F. (2019). Light pollution. In M. S. Charlesworth & A. C. Booth (Eds.), Urban pollution: Science and management (pp. 147–159). John Wiley and Sons.

[ece370018-bib-0040] Fattorini, S. (2011). Insect extinction by urbanization: A long term study in Rome. Biological Conservation, 144, 370–375. 10.1016/j.biocon.2010.09.014

[ece370018-bib-0041] Fattorini, S. (2020). Beetle species–area relationships and extinction rates in protected areas. Insects, 11, 646. 10.3390/insects11090646 32967143 PMC7563763

[ece370018-bib-0042] Fenger, J. (1999). Urban air quality. Atmospheric Environment, 33, 4877–4900. 10.1016/S13522310(99)00290-3

[ece370018-bib-0043] Flörke, H. (1815). Deutsche Lichenen, gesammelt und mit Anmerkungen herausgegeben .

[ece370018-bib-0044] Geissler, U. , & Kies, L. (2003). Artendiversität und Veränderungen in der Algenflora zweier städtischer Ballungsgebiete Deutschlands: Berlin und Hamburg. Nova Hedwigia, 126, 1–777.

[ece370018-bib-0045] Gelbrecht, J. , Kormannshaus, A. , Krüger, B. , Ockruck, F. , Schulze, B. , Theimer, F. , Weisbach, P. , Woelky, H. , Woelky, O. , & Woelky, M. (2022). Rote Liste und Gesamtartenliste der Großschmetterlinge (Lepidoptera: ‘Makrolepidoptera’) von Berlin. In: Der Landesbeauftragte für Naturschutz und Landschaftspflege / Senatsverwaltung für Umwelt, Mobilität, Verbraucher‐ und Klimaschutz, Landesfachausschuss Entomologie Berlin‐Brandenburg, NABU‐Landesverband Berlin (Eds.), Märkische Entomologische Nachrichten.

[ece370018-bib-0046] Github . (2023). Extinct species in Berlin . https://github.com/Zzzhenya/Berlin‐red‐list

[ece370018-bib-0047] Goddard, M. A. , Dougill, J. A. , & Benton, G. T. (2010). Scaling up from gardens: Biodiversity conservation in urban environments. Trends in Ecology and Evolution, 25, 90–98. 10.1016/j.tree.2009.07.016 19758724

[ece370018-bib-0048] Gottwald, S. (2017). Rote Liste und Gesamtartenliste der Prachtkäfer (Coleoptera: Buprestidae) von Berlin. In Der Landesbeauftragte für Naturschutz und Landschaftspflege/Senatsverwaltung für Umwelt, Verkehr und Klimaschutz (Ed.), Rote Listen der gefährdeten Pflanzen, Pilze und Tiere von Berlin. Technische Universität Berlin. 10.14279/depositonce-5854

[ece370018-bib-0049] Gregor, T. , Bönsel, D. , Starke‐Ottich, I. , & Zizka, G. (2012). Drivers of floristic change in large cities–A case study of Frankfurt/Main (Germany). Landscape and Urban Planning, 104, 230–237. 10.1016/j.landurbplan.2011.10.015

[ece370018-bib-0050] Haaland, C. , & van den Bosch, C. K. (2015). Challenges and strategies for urban green‐space planning in cities undergoing densification: A review. Urban Forestry and Urban Greening, 14, 760771. 10.1016/j.ufug.2015.07.009

[ece370018-bib-0051] Hackenberg, E. , & Müller, R. (2017). Rote Liste und Gesamtartenliste der Weichtiere (Mollusca: Gastropoda und Bivalvia) von Berlin. In Der Landesbeauftragte für Naturschutz und Landschaftspflege/Senatsverwaltung für Umwelt, Verkehr und Klimaschutz (Ed.), Rote Listen der gefährdeten Pflanzen, Pilze und Tiere von Berlin. Technische Universität Berlin. 10.14279/depositonce-5845

[ece370018-bib-0052] Hahs, A. K. , McDonnell, M. J. , McCarthy, M. A. , Vesk, P. A. , Corlett, R. T. , Norton, B. A. , Clemants, S. E. , Duncan, R. P. , Thompson, K. , Schwartz, M. W. , & Williams, N. S. G. (2009). A global synthesis of plant extinction rates in urban areas. Ecology Letters, 12, 1165–1173. 10.1111/j.1461-0248.2009.01372.x 19723284

[ece370018-bib-0053] Halley, J. M. , Sgardeli, V. , & Monokrousos, N. (2013). Species–area relationships and extinction forecasts. Annals of the New York Academy of Sciences, 1286, 50–61. 10.1111/nyas.12073 23672586

[ece370018-bib-0054] Hamburg in Zahlen . (2024). Fakten und Zahlen . https://www.hamburg.de/info/3277402/hamburg‐in‐zahlen/

[ece370018-bib-0055] Heinig, U. , & Schöller, M. (2017). Rote Liste und Gesamtartenliste der Blattkäfer (Coleoptera: Chrysomelidae und Megalopodidae) von Berlin. In Der Landesbeauftragte für Naturschutz und Landschaftspflege/Senatsverwaltung für Umwelt, Verkehr und Klimaschutz (Ed.), Rote Listen der gefährdeten Pflanzen, Pilze und Tiere von Berlin. Technische Universität Berlin. 10.14279/depositonce-5855

[ece370018-bib-0056] Heinrich, L. , & Müller, R. (2017). Rote Liste und Gesamtartenliste der Wasserkäfer von Berlin (Coleoptera: Hydradephaga, Hydrophiloidea part., Hydraenidae, Elmidae und Dryopidae). In Der Landesbeauftragte für Naturschutz und Landschaftspflege/Senatsverwaltung für Umwelt, Verkehr und Klimaschutz (Ed.), Rote Listen der gefährdeten Pflanzen, Pilze und Tiere von Berlin. Technische Universität Berlin. 10.14279/depositonce-5851

[ece370018-bib-0057] Hervé, M. (2013). Diverse basic statistical and graphical functions (RVAide memoire) . R package. https://CRAN.R‐project.org/package=RVAideMemoire

[ece370018-bib-0058] Hobbs, R. J. , Higgs, E. S. , & Hall, C. M. (2013). Novel ecosystems. Intervening in a new ecological world order. Wiley‐Blackwell.

[ece370018-bib-0059] Hochkirch, A. , Bilz, M. , Ferreira, C. C. , Danielczak, A. , Allen, D. , Nieto, A. , Rondinini, C. , Harding, K. , Hilton‐Taylor, C. , Pollock, C. M. , Seddon, M. , Vié, J. C. , Alexander, K. N. A. , Beech, E. , Biscoito, M. , Braud, Y. , Burfield, I. J. , Buzzetti, F. M. , Cálix, M. , … Zuna‐Kratky, T. (2023). A multitaxon analysis of European Red Lists reveals major threats to biodiversity. PLoS One, 18, e0293083. 10.1371/journal.pone.0293083 37939028 PMC10631624

[ece370018-bib-0060] IUCN . (2024). The IUCN Red Lists of Threatened Species. https://www.iucnredlist.org/

[ece370018-bib-0061] IUCN Standards and Petitions Committee . (2022). Guidelines for Using the IUCN Red List Categories and Criteria . Version 15.1. Prepared by the Standards and Petitions Committee. https://www.iucnredlist.org/documents/RedListGuidelines.pdf

[ece370018-bib-0062] IUCN Summary Statistics . (2023). Number of species in each IUCN Red List Category by kingdom and class . https://www.iucnredlist.org/statistics

[ece370018-bib-0063] Jablonski, D. (1986). Background and mass extinctions: The alternation of macroevolutionary regimes. Science, 231, 129–133. 10.1126/science.231.4734.129 17842630

[ece370018-bib-0064] Keinath, S. , Frisch, J. , Müller, J. , Mayer, F. , & Rödel, M.‐O. (2020). Spatio‐temporal color differences between urban and rural populations of a ground beetle during the last 100 years. Frontiers in Ecology and Evolution: Urban Ecology, 7, 525. 10.3389/fevo.2019.00525

[ece370018-bib-0065] Keinath, S. , Frisch, J. , Müller, J. , Mayer, F. , Struck, U. , & Rödel, M.‐O. (2023). Species‐ and sex‐dependent changes in body size between 1892 and 2017, and recent biochemical signatures in rural and urban populations of two ground beetle species. Ecology and Evolution, 13, e10329. 10.1002/ece3.10329 37484935 PMC10361362

[ece370018-bib-0066] Keinath, S. , Hölker, F. , Müller, J. , & Rödel, M.‐O. (2021). Impact of light pollution on moth morphology – A 137‐year study in Germany. Basic and Applied Ecology, 56C, 1–10. 10.1016/j.baae.2021.05.004

[ece370018-bib-0067] Kielhorn, K.‐H. (2005). Rote Liste und Gesamtartenliste der Laufkäfer (Coleoptera: Carabidae) von Berlin. In Der Landesbeauftragte für Naturschutz und Landschaftspflege/Senatsverwaltung für Umwelt, Verkehr und Klimaschutz (Ed.), Rote Listen der gefährdeten Pflanzen und Tiere von Berlin. Technische Universität Berlin.

[ece370018-bib-0068] Kielhorn, U. (2017). Rote Liste und Gesamtartenliste der Spinnen (Araneae) und Gesamtartenliste der Weberknechte (Opiliones) von Berlin. In Der Landesbeauftragte für Naturschutz und Landschaftspflege/Senatsverwaltung für Umwelt, Verkehr und Klimaschutz (Ed.), Rote Listen der gefährdeten Pflanzen, Pilze und Tiere von Berlin. Technische Universität Berlin. 10.14279/depositonce-5859

[ece370018-bib-0069] Klawitter, J. , Altenkamp, R. , Kallasch, C. , Köhler, D. , Krauß, M. , Rosenau, S. , & Teige, T. (2005). Rote Liste und Gesamtartenliste der Säugetiere (Mammalia) von Berlin. In Der Landesbeauftragte für Naturschutz und Landschaftspflege/Senatsverwaltung für Umwelt, Verkehr und Klimaschutz (Ed.), Rote Listen der gefährdeten Pflanzen und Tiere von Berlin. Technische Universität Berlin.

[ece370018-bib-0070] Klawitter, J. , & Köstler, H. (2017). Rote Liste und Gesamtartenliste der Moose (Bryophyta) von Berlin. In Der Landesbeauftragte für Naturschutz und Landschaftspflege/Senatsverwaltung für Umwelt, Verkehr und Klimaschutz (Ed.), Rote Listen der gefährdeten Pflanzen, Pilze und Tiere von Berlin. Technische Universität Berlin. 10.14279/depositonce-5844

[ece370018-bib-0071] Knapp, S. , Aronson, M. F. , Carpenter, E. , Herrera‐Montes, A. , Jung, K. , Kotze, D. J. , La Sorte, F. , Lepczyk, C. A. , MacGregor‐Fors, I. , Maclvor, J. S. , Moretti, M. , Nilson, C. H. , Piana, M. R. , Rega‐Brodsky, C. C. , Salisbury, A. , Threlfall, C. G. , Trisos, C. , Williams, N. S. G. , & Hahs, A. K. (2021). A research agenda for urban biodiversity in the global extinction crisis. Bioscience, 71, 268–279. 10.1093/biosci/biaa141

[ece370018-bib-0072] Korge, H. (2003). Rote Liste und Gesamtartenliste der Kurzflüfelkäfer (Coleoptera: Staphylinidae) von Berlin. In Der Landesbeauftragte für Naturschutz und Landschaftspflege/Senatsverwaltung für Umwelt, Verkehr und Klimaschutz (Ed.), Rote Listen der gefährdeten Pflanzen, Pilze und Tiere von Berlin. Technische Universität Berlin.

[ece370018-bib-0073] Korsch, H. , & Täuscher, L. (2016). Kurze Geschichte der Characeenkunde in Deutschland. In Arbeitsgruppe Characeen Deutschlands (Ed.), Armleuchteralgen. Die Characeen Deutschlands (pp. 5–15). Springer.

[ece370018-bib-0074] Krause, J. , Wagner, H.‐G. , & Otte, V. (2017). Rote Liste und Gesamtartenliste der Flechten (Lichenes) von Berlin. In Der Landesbeauftragte für Naturschutz und Landschaftspflege/Senatsverwaltung für Umwelt, Verkehr und Klimaschutz (Ed.), Rote Listen der gefährdeten Pflanzen, Pilze und Tiere von Berlin. Technische Universität Berlin. 10.14279/depositonce-5841

[ece370018-bib-0075] Kühn, I. , Brandl, R. , & Klotz, S. (2004). The flora of German cities is naturally species rich. Evolutionary Ecology Research, 6, 749–764.

[ece370018-bib-0076] Kühnel, K.‐D. , Scharon, J. , Kitzmann, B. , & Schonert, B. (2017a). Rote Liste und Gesamtartenliste der Lurche (Amphibia) von Berlin. In Der Landesbeauftragte für Naturschutz und Landschaftspflege/Senatsverwaltung für Umwelt, Verkehr und Klimaschutz (Ed.), Rote Listen der gefährdeten Pflanzen, Pilze und Tiere von Berlin. Technische Universität Berlin. 10.14279/depositonce-58

[ece370018-bib-0077] Kühnel, K.‐D. , Scharon, J. , Kitzmann, B. , & Schonert, B. (2017b). Rote Liste und Gesamtartenliste der Kriechtiere (Reptilia) von Berlin. In Der Landesbeauftragte für Naturschutz und Landschaftspflege/Senatsverwaltung für Umwelt, Verkehr und Klimaschutz (Ed.), Rote Listen der gefährdeten Pflanzen, Pilze und Tiere von Berlin. Technische Universität Berlin. 10.14279/depositonce-584

[ece370018-bib-0078] Kusber, W.‐H. , Jahn, R. , & Korsch, H. (2017). Rote Liste und Gesamtartenliste der Armleuchteralgen (Characeae) von Berlin. In Der Landesbeauftragte für Naturschutz und Landschaftspflege / Senatsverwaltung für Umwelt, Verkehr und Klimaschutz (Ed.), Rote Listen der gefährdeten Pflanzen, Pilze und Tiere von Berlin. Technische Universität Berlin. 10.14279/depositonce-5843

[ece370018-bib-0079] Le, X. D. (2008). Long‐term development of nutrient loads in Berlin surface water system and their causes during the last 150 years (Doctoral dissertation). 10.17169/refubium-16147

[ece370018-bib-0080] Lewis, D. (2018). Belonging on an Island: Birds, extinction, and evolution in Hawai‘i. Yale University Press. 10.12987/9780300235463

[ece370018-bib-0081] Liu, Z. , He, C. , & Wu, J. (2016). The relationship between habitat loss and fragmentation during urbanization: An empirical evaluation from 16 world cities. PLoS One, 11, e0154613. 10.1371/journal.pone.0154613 27124180 PMC4849762

[ece370018-bib-0082] Ludwig, G. , Haupt, H. , Gruttke, H. , & Binot‐Hafke, M. (2009). Methodik der Gefährdungsanalyse für Rote Listen. In H. Haupt , G. Ludwig , H. Gruttke , M. Binot‐Hafke , C. Otto , & A. Pauly (Eds.) Der Landesbeauftragte für Naturschutz und Landschaftspflege der Senatsverwaltung für Umwelt, Mobilität, Verbraucher‐ und Klimaschutz, Rote Liste gefährdeter Tiere, Pflanzen und Pilze Deutschlands, Volume 1: Wirbeltiere. Naturschutz und Biologische Vielfalt (Vol. 70, pp. 23–71).

[ece370018-bib-0083] Machatzi, B. , Ratsch, A. , Prasse, R. , & Ristow, M. (2005). Rote Liste und Gesamtartenliste der Heuschrecken und Grillen (Saltatoria: Ensifera et Caelifera) von Berlin. In Der Landesbeauftragte für Naturschutz und Landschaftspflege/Senatsverwaltung für Umwelt, Verkehr und Klimaschutz (Ed.), Rote Listen der gefährdeten Pflanzen und Tiere von Berlin. Technische Universität Berlin.

[ece370018-bib-0084] Maes, D. , & Van Dyck, H. (2001). Butterfly diversity loss in Flanders (north Belgium): Europe's worst case scenario? Biological Conservation, 99, 263–276. 10.1016/S0006-3207(00)00182-8

[ece370018-bib-0085] Magle, S. B. , Reyes, P. , Zhu, J. , & Crooks, K. R. (2010). Extirpation, colonization, and habitat dynamics of a keystone species along an urban gradient. Biological Conservation, 143, 2146–2155. 10.1016/j.biocon.2010.05.027

[ece370018-bib-0086] Marzluff, J. M. (2001). Worldwide urbanization and its effects on birds. In J. M. Marzluff , R. Bowman , & R. Donnelly (Eds.), Avian ecology in an urbanizing world (pp. 19–47). Kluwer.

[ece370018-bib-0087] McDonald, R. I. , Kareiva, P. , & Forman, R. T. T. (2008). The implications of current and future urbanization for global protected areas and biodiversity conservation. Biological Conservation, 141, 1695–1703. 10.1016/j.biocon.2008.04.025

[ece370018-bib-0088] McDonald, R. I. , Mansur, A. V. , Ascensão, F. , Colbert, M. L. , Crossman, K. , Elmqvist, T. , Gonzales, A. , Güneralp, B. , Haase, D. , Hemann, M. , Hillel, O. , Huang, K. , Kahnt, B. , Maddox, D. , Pacheco, A. , Pereira, H. M. , Seto, K. C. , Simkin, R. , Walsh, B. , … Ziter, C. (2020). Research gaps in knowledge of the impact of urban growth on biodiversity. Nature Sustainability, 3, 16–24. 10.1038/s41893-019-0436-6

[ece370018-bib-0089] McIntyre, N. E. (2000). Ecology of urban arthropods: a review and a call to action. Annals of the Entomological Society of America, 93, 825–835. 10.1603/0013-8746(2000)093[0825:EOUAAR]2.0.CO;2

[ece370018-bib-0090] McKinney, M. L. (2002). Urbanization, biodiversity, and conservation: The impacts of urbanization on native species are poorly studied, but educating a highly urbanized human population about these impacts can greatly improve species conservation in all ecosystems. Bio Science, 52, 883–890. 10.1641/0006-3568(2002)052[0883:UBAC]2.0.CO;2

[ece370018-bib-0091] McKinney, M. L. (2008). Effects of urbanization on species richness: A review of plants and animals. Urban Ecosystems, 11, 161–176. 10.1007/s11252-007-0045-4

[ece370018-bib-0092] McKinney, W. (2010). Data structures for statistical computing in python. In S. van der Walt ɬ J. Millman (Eds) Proceedings of the 9th Python in science conference (Vol. 445, pp. 51–56).

[ece370018-bib-0093] Mey, W. (2005). Rote Liste und Gesamtartenliste der Köcherfliegen (Trichoptera) von Berlin. In Der Landesbeauftragte für Naturschutz und Landschaftspflege/Senatsverwaltung für Stadtentwicklung (Ed.), Rote Listen der gefährdeten Pflanzen und Tiere von Berlin. Technische Universität Berlin.

[ece370018-bib-0094] Müller, R. (2017). Rote Liste und Gesamtartenliste der Eintagsfliegen (Ephemeroptera) von Berlin. In Der Landesbeauftragte für Naturschutz und Landschaftspflege/Senatsverwaltung für Umwelt, Verkehr und Klimaschutz (Ed.), Rote Listen der gefährdeten Pflanzen, Pilze und Tiere von Berlin. Technische Universität Berlin. 10.14279/depositonce-5848

[ece370018-bib-0095] Müller, R. , & Mey, W. (2017). Rote Liste und Gesamtartenliste der Köcherfliegen (Trichoptera) von Berlin. In Der Landesbeauftragte für Naturschutz und Landschaftspflege/Senatsverwaltung für Umwelt, Verkehr und Klimaschutz (Ed.), Rote Listen der gefährdeten Pflanzen, Pilze und Tiere von Berlin. Technische Universität Berlin. 10.14279/depositonce-5857

[ece370018-bib-0096] Nazarevich, V. J. (2015). The sixth species extinction event by humans. Earth Common Journal, 5, 61–72. 10.31542/j.ecj.261

[ece370018-bib-0097] Nickel, H. , & Mühlethaler, R. (2017). Rote Liste und Gesamtartenliste der Zikaden (Hemiptera: Fulgoromorpha und Cicadomorpha) von Berlin. In Der Landesbeauftragte für Naturschutz und Landschaftspflege/Senatsverwaltung für Umwelt, Verkehr und Klimaschutz (Ed.), Rote Listen der gefährdeten Pflanzen, Pilze und Tiere von Berlin. Technische Universität Berlin. 10.14279/depositonce-5850

[ece370018-bib-0098] Niederländischer Landesbetrieb für Wasserwirtschaft, Küsten‐ und Naturschutz [NLWKN] . (2024). Rote Listen . https://www.nlwkn.niedersachsen.de/naturschutz/tier_und_pflanzenartenschutz/rote_listen/rote‐listen‐46118.html

[ece370018-bib-0099] Niedersachsen . (2024). Niedersachsen in Zahlen . https://www.niedersachsen.de/startseite/land_leute/das_land/zahlen_fakten/niedersachsen‐in‐zahlen‐20094.html#:~:text=Mit%20rund%2047%20614%20km%C2%B2,der%20Bev%C3%B6lkerungszahl%20nach%20viertgr%C3%B6%C3%9Fte%20Bundesland

[ece370018-bib-0100] Niemeier, S. , Müller, J. , Struck, U. , & Rödel, M.‐O. (2020). Superfrogs in the city: 150 year impact of urbanization and agriculture on the European Common frog. Global Change Biology, 26, 6729–6741. 10.1111/gcb.15337 32975007

[ece370018-bib-0101] Pamme, H. (2003). Das Politikfeld Umweltpolitik. In D. Grunow (Ed.), Verwaltungshandeln in Politikfeldern (pp. 185–224). Leske and Budrich.

[ece370018-bib-0102] Pandas . (2024). Pandas shape type: Pandas . DataFrame.shape. https://pandas.pydata.org/docs/reference/api/pandas.DataFrame.shape.html

[ece370018-bib-0103] Petzold, F. (2017). Rote Liste und Gesamtartenliste der Libellen (Odonata) von Berlin. In Der Landesbeauftragte für Naturschutz und Landschaftspflege/Senatsverwaltung für Umwelt, Verkehr und Klimaschutz (Ed.), Rote Listen der gefährdeten Pflanzen, Pilze und Tiere von Berlin. Technische Universität Berlin. 10.14279/depositonce-5849

[ece370018-bib-0104] Platen, R. , & von Broen, B. (2005). Gesamtartenliste und Rote Liste der Webspinnen und Weberknechte (Arachnida: Araneae, Opiliones) des Landes Berlin. In Der Landesbeauftragte für Naturschutz und Landschaftspflege/Senatsverwaltung für Stadtentwicklung (Ed.), Rote Listen der gefährdeten Pflanzen und Tiere von Berlin. Technische Universität Berlin.

[ece370018-bib-0105] Prasse, R. , Ristow, M. , Klemm, G. , Machatzi, B. , Raust, T. , Scholz, H. , Stohr, G. , Sukopp, H. , & Zimmermann, F. (2001). Liste der wildwachsenden Gefäßpflanzen des Landes Berlin mit Roter Liste. In Senatsverwaltung für Stadtentwicklung, Der Landesbeauftragte für Naturschutz und Landschaftspflege (Ed.), Rote Listen der gefährdeten Pflanzen und Tiere von Berlin. Technische Universität Berlin.

[ece370018-bib-0106] Project Jupyter . (2016). JupyterLab software – Code version: 4.0.6 . https://jupyterlab.readthedocs.io/

[ece370018-bib-0107] Purvis, A. , Jones, E. K. , & Mace, M. G. (2000). Extinction. BioEssays, 22, 1123–1133. 10.1002/1521-1878(200012)22:12<1123::AID-BIES10>3.0.CO;2-C 11084628

[ece370018-bib-0108] Pyšek, P. , Hulme, P. E. , Simberloff, D. , Bacher, S. , Blackburn, T. M. , Carlton, J. T. , Dawson, W. , Essl, F. , Foxcroft, L. C. , Genovesi, P. , Jeschke, J. M. , Kühn, I. , Liebhold, A. M. , Mandrak, N. E. , Meyerson, L. A. , Pauchard, A. , Pergl, J. , Roy, H. E. , Seebens, H. , … Richardson, D. M. (2020). Scientists' warning on invasive alien species. Biological Reviews, 95, 1511–1534. 10.1111/brv.12627 32588508 PMC7687187

[ece370018-bib-0109] R Core Team . (2023). R: A language and environment for statistical computing. R Foundation for Statistical Computing. https://www.R‐project.org/

[ece370018-bib-0110] Ramankutty, N. , Mehrabi, Z. , Waha, K. , Jarvis, L. , Kremen, C. , Herrero, M. , & Rieseberg, L. H. (2018). Trends in global agricultural land use: Implications for environmental health and food security. Annual Review of Plant Biology, 69, 789–815. 10.1146/annurev-arplant-042817-040256 29489395

[ece370018-bib-0111] Raup, D. M. (1994). The role of extinction in evolution. Proceedings of National Academy of Sciences of the United States of America, 91, 6758–6763. 10.1073/pnas.91.15.6758 PMC44280 8041694

[ece370018-bib-0112] Reineck, G. (1919). Die Insekten der Mark Brandenburg. Volume 2 Cerambycidae. Beiheft der Deutschen Entomologischen Gesellschaft (pp. 1–92).

[ece370018-bib-0113] Ribbe, W. , Bohm, E. , Schich, W. , & Schulz, K. (2002a). Geschichte Berlins. Volume 1: Von der Frühgeschichte bis zur Industrialisierung. Beck.

[ece370018-bib-0114] Ribbe, W. , Bohm, E. , Schich, W. , & Schulz, K. (2002b). Geschichte Berlins. Volume 2: Von der Märzrevolution bis zur Gegenwart. Beck.

[ece370018-bib-0115] Riecken, U. , Binot‐Hafke, M. , Gruttke, H. , Korneck, D. , & Ludwig, G. (2000). Fortschreitung und Perspektiven von bundesweiten Roten Listen. Schriftreihe für Landschaftspflege und Naturschutz, 65, 231–255.

[ece370018-bib-0116] Ring, P. (1992). Bevölkerung. Berlin Handbuch (pp. 237).

[ece370018-bib-0117] Rissberger, S. (1985). On the brink of an ecological calamity: Acid rain, transboundary air pollution and environmental law in West Germany. Syracuse Journal of International Law and Commerce, 12, 325.

[ece370018-bib-0118] Rizwan, A. M. , Dennis, L. Y. C. , & Liu, C. (2008). A review on the generation, determination and mitigation of urban heat Island. Journal of Ecology and Environmental Science, 20, 120–128. 10.1016/S1001-0742(08)60019-4 18572534

[ece370018-bib-0119] Rote Liste Saarland . (2024). Pflanzen‐ und Tierarten des Saarlandes – Bestand und Gefährdung . https://rote‐liste‐saarland.de/

[ece370018-bib-0120] Rote Liste Zentrum . (2024a). Die Roten Listen . https://www.rote‐liste‐zentrum.de/de/Die‐Roten‐Listen‐1707.html

[ece370018-bib-0121] Rote Liste Zentrum . (2024b). Vergleich mit anderen Roten Listen . https://www.rote‐liste‐zentrum.de/de/Vergleich‐mit‐anderen‐Roten‐Listen‐1713.html#:~:text=Rote%20Listen%20der%20Bundesl%C3%A4nder,‐Mehr%20erfahren%20auf&text=Baden%2DW%C3%BCrttemberg%2C%20Bayern%2C%20Berlin,%2C%20Schleswig%2DHolstein%2C%20Th%C3%BCringen

[ece370018-bib-0122] Roy, H. E. , Pauchard, A. , Stoett, P. , Truong, T. R. , Lipinskaya, T. , & Vincete, J. R. (2023). ). IPBES Invasive Alien Species Assessment: Chapter 1. Introducing biological invasions and the IPBES thematic assessment of invasive alien species and their control. *Zenodo*. 10.5281/zenodo.10056589

[ece370018-bib-0123] Rudolph, K. , Jahn, R. , & Kusber, W.‐H. (2017). Rote Liste und Gesamtartenliste der limnischen Rotalgen (Rhodophyta) und Braunalgen (Phaeophyceae) von Berlin. In Der Landesbeauftragte für Naturschutz und Landschaftspflege / Senatsverwaltung für Umwelt, Verkehr und Klimaschutz (Ed.), Rote Listen der gefährdeten Pflanzen, Pilze und Tiere von Berlin. 10.14279/depositonce5842

[ece370018-bib-0124] Saarland . (2022). Saarland kompakt . https://www.saarland.de/DE/land‐leute/saarland‐kompakt#:~:text=Das%20Saarland.,Est%2C%20nach%20Westen%20an%20Luxemburg

[ece370018-bib-0125] Saure, C. (2000). Rote Liste und Artenliste der Bienen des Landes Brandenburg (Hymenoptera: Apidae). Naturschutz und Landschaftspflege in Brandenburg, 9, 1.

[ece370018-bib-0126] Saure, C. (2005a). Rote Liste und Gesamtartenliste der Kamelhalsfliegen, Schlammfliegen und Netzflügler (Raphidioptera, Megaloptera, Neuroptera) von Berlin. In Der Landesbeauftragte für Naturschutz und Landschaftspflege/Senatsverwaltung für Umwelt, Verkehr und Klimaschutz (Ed.), Rote Listen der gefährdeten Pflanzen und Tiere von Berlin. Technische Universität Berlin.

[ece370018-bib-0127] Saure, C. (2005b). Rote Liste und Gesamtartenliste der Bienen und Wespen (Hymenoptera part.) von Berlin mit Angaben zu den Ameisen. In Der Landesbeauftragte für Naturschutz und Landschaftspflege/Senatsverwaltung für Umwelt, Verkehr und Klimaschutz (Ed.), Rote Listen der gefährdeten Pflanzen und Tiere von Berlin. Technische Universität Berlin.

[ece370018-bib-0128] Saure, C. (2005c). Rote Liste und Gesamtartenliste der Schnabelfliegen (Mecoptera) von Berlin. In Der Landesbeauftragte für Naturschutz und Landschaftspflege/Senatsverwaltung für Umwelt, Verkehr und Klimaschutz (Ed.), Rote Listen der gefährdeten Pflanzen und Tiere von Berlin. Technische Universität Berlin.

[ece370018-bib-0129] Saure, C. (2018). Rote Liste und Gesamtartenliste der Schwebfliegen (Diptera: Syrphidae) von Berlin. In Der Landesbeauftragte für Naturschutz und Landschaftspflege/Senatsverwaltung für Umwelt, Verkehr und Klimaschutz (Ed.), Rote Listen der gefährdeten Pflanzen, Pilze und Tiere von Berlin. Technische Universität Berlin. 10.14279/depositonce-6691

[ece370018-bib-0130] Saure, C. , Burger, F. , & Dathe, H. H. (1999). Die Bienenarten von Brandenburg und Berlin (Hymenoptera: Apidae). Entomologische Nachrichten und Berichte, 42, 155–166.

[ece370018-bib-0131] Saure, C. , Burger, F. , & Oehlke, J. (1998). Rote Liste und Artenliste der Gold‐, Falten‐ und Wegwespen des Landes Brandenburg (Hymenoptera: Chrysididae, Vespidae, Pompilidae). Landesumweltamt Brandenburg. Naturschutz und Landschaftspflege in Brandenburg, 7, 2.

[ece370018-bib-0132] Saure, C. , & Kielhorn, K.‐H. (2005). Rote Listen der gefährdeten Pflanzen und Tiere von Berlin – Zusammenfassung und Bilanz. In Der Landesbeauftragte für Naturschutz und Landschaftspflege / Senatsverwaltung für Umwelt, Verkehr und Klimaschutz (Ed.), Rote Listen der gefährdeten Pflanzen und Tiere von Berlin. Technische Universität Berlin.

[ece370018-bib-0133] Saure, C. , & Schwarz, J. (2005). Methodische Grundlagen. In Der Landesbeauftragte für Naturschutz und Landschaftspflege/Senatsverwaltung für Stadtentwicklung (Ed.), Rote Listen der gefährdeten Pflanzen und Tiere von Berlin. Technische Universität Berlin.

[ece370018-bib-0134] Schalow, H. (1919). Beiträge zur Vogelfauna der Mark Brandenburg: Materialien zu einer Ornithologie der norddeutschen Tiefebene auf Grund eigener Beobachtungen und darauf gegründeter Studien. Deutsche Ornithologische Gesellschaft.

[ece370018-bib-0135] Schewenius, M. , McPhearson, T. , & Elmqvist, T. (2014). Opportunities for increasing resilience and sustainability of urban social–ecological systems: Insights from the URBES and the cities and biodiversity outlook projects. Ambio, 43, 434–444. 10.1007/s13280-014-0505-z 24740615 PMC3989512

[ece370018-bib-0136] Schildt, A. , & Sywottek, A. (1993). Modernisierung im Wiederaufbau ‐ Die westdeutsche Gesellschafft der 50er Jahre. Verlag J.H.W. Dietz.

[ece370018-bib-0137] Schirmer, C. (1912). Weitere Beiträge zur Kenntnis der Insekten der Mark Brandenburg. *Neuroptera genuina*. Gruppe II Planipennia. Archiv für Naturgeschichte, 78, 137–140.

[ece370018-bib-0138] Schmidt, M. (2017). Rote Liste und Gesamtartenliste der Röhrlinge s. l. (Boletales) von Berlin. In Der Landesbeauftragte für Naturschutz und Landschaftspflege/Senatsverwaltung für Umwelt, Verkehr und Klimaschutz (Ed.), Rote Listen der gefährdeten Pflanzen, Pilze und Tiere von Berlin. Technische Universität Berlin. 10.14279/depositonce-5839

[ece370018-bib-0139] Schmidt, M. , & Täglich, U. (2023). Rote Liste und Gesamtartenliste der Schleimpilze (Myxomycetes inkl. Ceratiomyxomycetes) von Berlin. In Der Landesbeauftragte für Naturschutz und Landschaftspflege/Senatsverwaltung für Mobilität, Verkehr, Klimaschutz und Umwelt (Ed.), Rote Listen der gefährdeten Pflanzen, Pilze und Tiere von Berlin. Technische Universität Berlin.

[ece370018-bib-0140] Schnittler, M. , Ludwig, G. , Pretscher, P. , & Boye, P. (1994). Konzeption der Roten Listen der in Deutschland gefährdeten Tier‐ und Pflanzenarten – unter Berücksichtigung der neuen internationalen Kategorien. Natur und Landschaft, 69, 451–459.

[ece370018-bib-0141] Scholz, H. , & Scholz, I. (2005). Rote Liste und Gesamtartenliste der Brandpilze (Ustilaginales) von Berlin. In Der Landesbeauftragte für Naturschutz und Landschaftspflege/Senatsverwaltung für Umwelt, Verkehr und Klimaschutz (Ed.), Rote Listen der gefährdeten Pflanzen und Tiere von Berlin. Technische Universität Berlin.

[ece370018-bib-0142] Seitz, B. , Ristow, M. , Meißner, J. , Machatzi, B. , & Sukopp, H. (2018). Rote Liste und Gesamtartenliste der etablierten Farn‐ und Blütenpflanzen von Berlin. In Der Landesbeauftragte für Naturschutz und Landschaftspflege/Senatsverwaltung für Umwelt, Verkehr und Klimaschutz (Ed.), Rote Listen der gefährdeten Pflanzen, Pilze und Tiere von Berlin. Technische Universität Berlin. 10.14279/depositonce-6689

[ece370018-bib-0143] Seitz, B. , Ristow, M. , Prasse, R. , Machatzi, B. , Klemm, G. , Böcker, R. , & Sukopp, H. (2012). Der Berliner Florenatlas. Verhandlungen des Botanischen Vereins von Berlin und Brandenburg, 7, 533.

[ece370018-bib-0144] Senatsverwaltung für Mobilität, Verkehr, Klimaschutz und Umwelt [SenMVKU] . (2024a). Berlin – Artenlisten – Rote Listen der gefährdeten Pflanzen, Tiere und Pilze von Berlin . https://www.berlin.de/sen/uvk/natur‐und‐gruen/naturschutz/artenschutz/artenlisten‐rote‐listen/

[ece370018-bib-0145] Senatsverwaltung für Mobilität, Verkehr, Klimaschutz und Umwelt, Berlin [SenMVKU] . (2024b). Natur und Grün . https://www.berlin.de/sen/uvk/natur‐und‐gruen/

[ece370018-bib-0146] Senatsverwaltung für Mobilität, Verkehr, Klimaschutz und Umwelt [SenMVKU] . (2024c). Berlin – Stadtbäume . https://www.berlin.de/sen/uvk/natur‐und‐gruen/stadtgruen/daten‐und‐fakten/stadtbaeume/

[ece370018-bib-0147] Senatsverwaltung für Umwelt, Verkehr und Klimaschutz [SenUVK] . (2019). Fische in Berlin – Bilanz der Artenvielfalt. Allgemeiner Teil.

[ece370018-bib-0148] Senatsverwaltung Stadtentwicklung (SenStadt) . (2020). Bauen und Wohnen (2020). Berliner Umweltatlas. Grün und Freiflächenbestand . https://www.berlin.de/umweltatlas/nutzung/flaechennutzung/2020/kartenbeschreibung/

[ece370018-bib-0149] SenMVKU . (2014). Senatsverwaltung für Mobilität, Verkehr, Klimaschutz und Umwelt Berlin, Germany. Fische in Berlin – Bilanz der Artenvielfalt. Senatsverwaltung für Stadtentwicklung und Umwelt.

[ece370018-bib-0150] Seto, K. C. , Güneralp, B. , & Hutyra, L. R. (2012). Global forecasts of urban expansion to 2030 and direct impacts on biodiversity and carbon pools. Proceedings of the National Academy of Science of the United States of America, 109, 16083–16088. 10.1073/pnas.1211658109 PMC3479537 22988086

[ece370018-bib-0151] Singh, A. , & Agrawal, M. (2008). Acid rain and its ecological consequences. Journal of Environmental Biology, 29, 15–24. 18831326

[ece370018-bib-0152] Smith‐Patten, B. , Bridge, S. E. , Crawford, C. H. P. , Hough, J. D. , Kelly, F. J. , & Patten, A. M. (2015). Is extinction forever? Public Understanding of Science, 24, 481–495. 10.1177/0963662515571489 25711479 PMC4404403

[ece370018-bib-0153] Sodhi, N. S. , Brook, B. W. , & Bradshaw, C. J. (2009). Causes and consequences of species extinctions. The Princeton Guide to Ecology, 1, 514–520. 10.1515/9781400833023.514

[ece370018-bib-0154] Sommerwerk, N. , Geschke, J. , Schliep, R. , Esser, J. , Glöckler, F. , Grossert, H.‐P. , Hand, R. , Kierfer, S. , Kimming, S. , Koch, A. , Kühn, E. , Larondelle, N. , Lehmann, G. , Munzinger, S. , Rödl, T. , Werner, D. , Wessel, M. , & Vohland, K. (2021). Vernetzung und Kooperation ehrenamtlicher und akademischer Forschung im Rahmen des internationalen Biodiversitätsmonitoring – Herausforderungen und Lösungsstrategien. Naturschutz und Landschaftsplanung, 53, 8. 10.1399/NuL.2021.08.03

[ece370018-bib-0155] Soundranayagam, J. P. , Sivasubramanian, P. , Chandrasekar, N. , & Duraira, K. S. P. (2011). An analysis of land use pattern in the industrial development city using high resolution satellite imagery. Journal of Geographical Sciences, 21, 79–88. 10.1007/s11442-011-0830-0

[ece370018-bib-0156] Speyer, A. D. , & Speyer, A. U. (1862). Die geographische Verbreitung der Schmetterlinge Deutschlands und der Schweiz. Volume 2: Die Noctuiden im weiteren Sinne (320 p).

[ece370018-bib-0157] Statista . (2023). Bevölkerung von Berlin . https://de.statista.com/statistik/daten/studie/154880/umfrage/entwicklung‐der‐bevoelkerung‐von‐berlin‐seit‐1961/

[ece370018-bib-0158] Statista . (2024a). Urbanisierungsgrad: Anteil der Stadtbewohner an der Gesamtbevölkerung in Deutschland in den Jahren von 2000 bis 2022 . https://de.statista.com/statistik/daten/studie/662560/umfrage/urbanisierung‐in‐deutschland/#:~:text=Die%20Urbanisierung%20bezeichnet%20den%20Anteil,der%20Gesamtbev%C3%B6lkerung%20Deutschlands%20in%20St%C3%A4dten

[ece370018-bib-0159] Statista . (2024b). Anteil von Stadt‐ und Landbewohnern in Deutschland von 1990 bis 2015 und Prognose bis 2050 . https://de.statista.com/statistik/daten/studie/167166/umfrage/prognose‐des‐bewohneranteils‐nach‐wohnstandort‐seit‐1990/#:~:text=Im%20Jahr%202050%20werden%20nach,Gesamtbev%C3%B6lkerung%20Deutschlands%20in%20St%C3%A4dten%20wohnen

[ece370018-bib-0160] Statista . (2024c). Entwicklung der Fläche der Stadt Berlin von 1640 bis 2019 . https://de.statista.com/statistik/daten/studie/657678/umfrage/flaeche‐der‐stadt‐berlin/

[ece370018-bib-0161] Statista . (2024d). Mittlerer Versieglungsgrad der größten in Deutschland (Stand: 2023) . https://de.statista.com/statistik/daten/studie/1419122/umfrage/versiegelungsgrad‐der‐groessten‐staedte‐in‐deutschland/

[ece370018-bib-0162] Statistische Ämter . (2024). Des Bundes und der Länder. Gemeinsames Statistikportal. Versiegelte Fläche 2016‐2022 nach Bundesländern . https://www.statistikportal.de/de/ugrdl/ergebnisse/flaeche‐und‐raum/vf

[ece370018-bib-0163] Statistische Jahrbücher der Stadt Berlin . (1920, 1924–1998, 2000). Statistischen Landesamt Berlin .

[ece370018-bib-0164] Steffen, W. , Grinevald, J. , Crutzen, P. , & McNeill, J. (2011). The Anthropocene: Conceptual and historical perspectives. Philosophical Transactions of the Royal Society, 369, 842–868. 10.1098/rsta.2010.0327 21282150

[ece370018-bib-0165] Stein, J. P. E. F. (1863). Beitrag zur Neuropteren‐Fauna Griechenlands. Berliner Entomologische Zeitschrift, 8, 411–441.

[ece370018-bib-0166] Strübing, H. (1956). Beiträge zur Ökologie einiger Hochmoorzikaden (Homoptera, Auchenorrhyncha). Österreichische Zoologische Zeitschrift, 6, 566–596.

[ece370018-bib-0167] Suhonen, J. , Korkeamäki, E. S. A. , Salmela, J. , & Kuitunen, M. (2014). Risk of local extinction of Odonata freshwater habitat generalists and specialists. Conservation Biology, 28, 783–789. 10.1111/cobi.12231 24405332

[ece370018-bib-0168] Sukopp, H. , & Elvers, H. (1982). Rote Listen der gefährdeten Pflanzen und Tiere in Berlin (West). Landschaftsentwicklung und Umweltforschung, 11, 374.

[ece370018-bib-0169] Theng, M. , Jusoh, W. F. , Jain, A. , Huertas, B. , Tan, D. J. , Tan, H. Z. , Kristensen, N. P. , Meier, R. , & Chisholm, R. A. (2020). A comprehensive assessment of diversity loss in a well‐documented tropical insect fauna: Almost half of Singapore's butterfly species extirpated in 160 years. Biological Conservation, 242, 108401. 10.1016/j.biocon.2019.108401

[ece370018-bib-0170] Theodorous, P. (2022). The effects of urbanisation on ecological interactions. Current Opinion in Insect Science, 52, 100922. 10.1016/j.cois.2022.100922 35490874

[ece370018-bib-0171] Tilman, D. , Cassman, K. G. , Matson, A. P. , Naylor, R. , & Polasky, S. (2002). Agricultural sustainability and intensive production practices. Nature, 418, 671–677. 10.1038/nature01014 12167873

[ece370018-bib-0172] Turnhout, E. , & Purvis, A. (2020). Biodiversity and species extinction: Categorisation, calculation, and communication. Griffith Law Review, 29, 669–685. 10.1080/10383441.2020.1925204

[ece370018-bib-0173] UBA . (1998). Umweltdaten Deutschland. Umweltbundesamt, Erich Schmidt Verlag.

[ece370018-bib-0174] Umweltatlas Berlin . (2020). Freiflächenentwicklung 2020 . https://www.berlin.de/umweltatlas/nutzung/freiflaechenentwicklung/fortlaufend‐aktualisiert/einleitung/

[ece370018-bib-0175] Umweltatlas Berlin . (2021). Versiegelung . https://www.berlin.de/umweltatlas/boden/versiegelung/

[ece370018-bib-0176] UNEP/WHO . (1993). City Air Quality Trends, Volume 2. WHO PEP/93 26, UNE93 26P GEMS 93.A.2, Genf .

[ece370018-bib-0177] United Nations . (2019). Department of Economic and Social Affairs, Population Division. World Urbanization Prospects: The 2018 revision (ST/ESA/SER.A/420). United Nations.

[ece370018-bib-0178] United Nations . (2022). Department of Economic and Social Affairs, Population Division. *World POPULATION PROSPECTS: Summary of results*. UN DESA/POP/2022/TR/NO. 3.

[ece370018-bib-0179] Van Rossum, G. , & Drake, F. L. (2009). Python 3 reference manual. CreateSpace.

[ece370018-bib-0180] Van't Hof, A. , Edmonds, E. , Dalikova, M. , Marec, F. , & Saccheri, J. I. (2011). Industrial melanism in British Peppered Moths has a singular and recent mutational origin. Science, 332, 958–960. 10.1126/science.1203043 21493823

[ece370018-bib-0181] Vinayak Meta . (2023). Title of software program: Camelot‐py code version: 0.11.0 . https://camelot‐py.readthedocs.io/en/master/

[ece370018-bib-0182] Wagner, H.‐G. , Krause, J. , & Otte, V. (2017). Rote Liste und Gesamtartenliste der flechtenbewohnenden (lichenicolen) Pilze von Berlin. In Der Landesbeauftragte für Naturschutz und Landschaftspflege/Senatsverwaltung für Umwelt, Verkehr und Klimaschutz (Ed.), Rote Listen der gefährdeten Pflanzen, Pilze und Tiere von Berlin. Technische Universität Berlin. 10.14279/depositonce-5840

[ece370018-bib-0183] Wanach, B. (1915). Die Neuropterenfauna Potsdams. Deutsche Entomologische Zeitschrift, 1915, 323–325.

[ece370018-bib-0184] Weidlich, M. (2022). Rote Liste und Gesamtartenliste der Sackträger (Lepidoptera: Psychidae) von Berlin. Märkische Entomologische Nachrichten, 1, 109–122.

[ece370018-bib-0185] Wey, K.‐G. (1982). Umweltpolitik in Deutschland. Kurze Geschichte des Umweltschutzes in Deutschland seit 1900. Westdeutscher Verlag.

[ece370018-bib-0186] Wiens, D. , Michéle, R. , & Slaton, R. M. (2012). The mechanism of background extinction. Biological Journal of the Linnean Society, 105, 255–268. 10.1111/j.1095-8312.2011.01819.x

[ece370018-bib-0187] Williams, N. S. , Morgan, J. W. , McDonnell, M. J. , & Mccarthy, M. A. (2005). Plant traits and local extinctions in natural grasslands along an urban–rural gradient. Journal of Ecology, 93, 1203–1213. 10.1111/j.1365-2745.2005.01039.x

[ece370018-bib-0188] Witt, K. , & Steiof, K. (2013). Rote Liste der Brutvögel von Berlin. Berliner Ornithologischer Bericht, 23, 1–23.

[ece370018-bib-0189] Witte, F. , Goldschmidt, T. , Goudswaard, P. C. , Ligtvoet, W. , Van Oijen, M. J. P. , & Wanink, J. (1992). Species extinction and concomitant ecological changes in Lake Victoria. Netherlands Journal of Zoology, 42, 214–232.

[ece370018-bib-0190] Wolff, D. (1998). Zur Schwebfliegenfauna des Berliner Raums (Diptera, Syrphidae). Volucella, 3, 87–131.

[ece370018-bib-0191] Wollmuth, E. M. , Sless, T. J. L. , Airey, M. E. , France, E. D. , Stump, E. M. , Sundstrom, M. A. , Wilkins, R. L. , & Smith, M. K. (2022). Is Earth currently undergoing a sixth mass extinction? CourseSource, 9, 1–10. 10.24918/cs.2022.19

[ece370018-bib-0192] Zuur, A. , Ieno, E. N. , Walker, N. , Saveliev, A. A. , & Smith, F. G. M. (2009). Mixed effects models and extensions in ecology with R. Springer.

