# Supplementary material for: High levels of species' extirpation in an urban environment—A case study from Berlin, Germany, covering 1700–2023
Source: Ecol Evol. 2024 Jul 15;14(7):e70018. doi: 10.1002/ece3.70018 (PMC11250399; doi:10.1002/ece3.70018)
Supplement: Supplementary file 1 — Appendix S1 [file ECE3-14-e70018-s003.zip › ece370018-sup-0001-Legend.docx]

**Appendix S1:**

Figure A1: Percentage of species per Red Lists of Threatened Species of the federal state of Berlin in the Red List categories: Unthreatened indigenous species (blue), species on the prewarning list (skin tone), endangered species (category 3, yellow), highly endangered species (category 2, orange), species threatened by extinction (category 1, light red), extinct or extirpated species (category 0, dark red).
