# Supplementary figures and images for: High levels of species' extirpation in an urban environment—A case study from Berlin, Germany, covering 1700–2023
Source: Ecol Evol. 2024 Jul 15;14(7):e70018. doi: 10.1002/ece3.70018 (PMC11250399; doi:10.1002/ece3.70018)

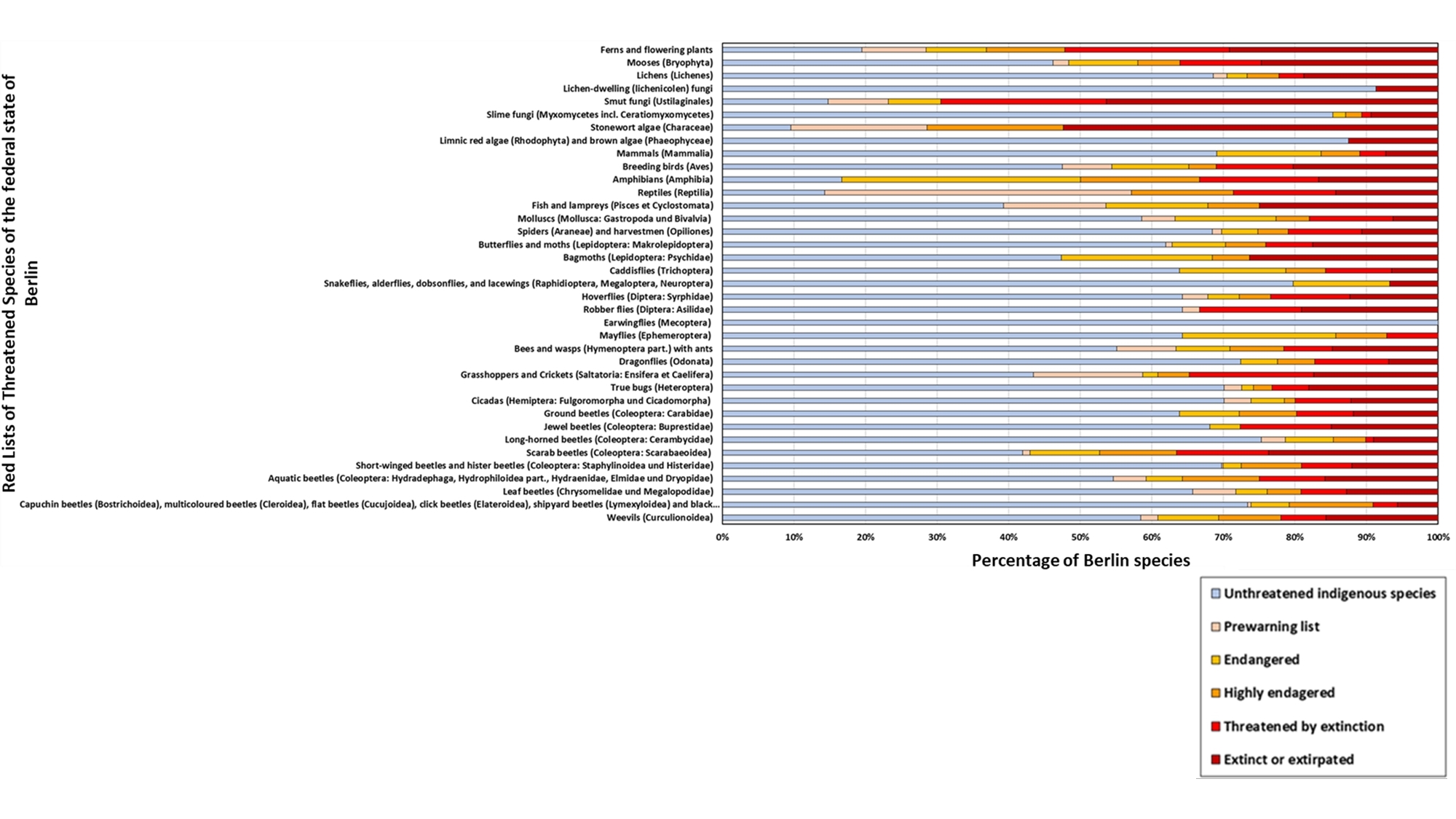

Supplement: Supplementary file 1 — Appendix S1 [file ECE3-14-e70018-s003.zip › ece370018-sup-0001-AppendixS1.jpg]
