# Supplementary material for: High levels of species' extirpation in an urban environment—A case study from Berlin, Germany, covering 1700–2023
Source: Ecol Evol. 2024 Jul 15;14(7):e70018. doi: 10.1002/ece3.70018 (PMC11250399; doi:10.1002/ece3.70018)
Supplement: Supplementary file 2 — Appendix S2 [file ECE3-14-e70018-s002.docx]

Appendix S2

Table B1: Summary statistics of pairwise G-test with Bonferroni correction for multiple testing for numbers of extirpated species between the 17th, 18th, 19th, 20th, and 21th centuries.

| **Group of comparison** | | **p-value** |
| --- | --- | --- |
| 17th century | 18th century | 1 |
| 17th century | 19th century | < 0.001 |
| 17th century | 20th century | < 0.001 |
| 17th century | 21th century | < 0.001 |
| 18th century | 19th century | < 0.001 |
| 18th century | 20th century | < 0.001 |
| 18th century | 21th century | < 0.001 |
| 19th century | 20th century | < 0.001 |
| 19th century | 21th century | < 0.001 |
| 20th century | 21th century | < 0.001 |

Table B2: Overview of the time windows the 37 Red Lists of Threatened Species of the federal state of Berlin look back.

| **Taxonomic group** | **Evidence on the reference time window** | **Century** | **Reference** |
| --- | --- | --- | --- |
| Ferns and flowering plants | Since the 18th century from the Berliner Florenatlas | 18th century | Seitz et al. (2012) |
| Mosses (Bryophyta) | Since the end of 18th century | 18th century | Klawitter and Köstler (2017) |
| Lichens (Lichens) | Since the beginning of the 19th century | 19th century | Flörke (1815) |
| Lichen-dwelling (lichenicolen) fungi | Since the beginning of the 19th century | 19th century | Flörke (1815) |
| Smut fungi (Ustilaginales) | Before 1945 | 20th century | Scholz and Scholz (2003) |
| Slime fungi (Myxomycetes incl. Ceratiomyxomycetes) | Since 1823 | 19th century | Ehrenberg (1818) |
| Stonewort algae (Characeae) | Since the 18th century | 18th century | Korsch and Täuscher (2016) |
| Limnic red algae (Rhodophyta) and brown algae (Phaeophyceae) | Since the late 19th century | 19th century | Geissler and Kies (2003) |
| Mammals (Mammalia) | Mammals that probably also occurred in Berlin before 1927 are not taken into account | 19th century | Klawitter et al. (2005) |
| Breeding birds (Aves) | Since the turn of the 19th and 20th century | 19th to 20th century | Schalow (1919) |
| Amphibians (Amphibia) | From 1920 on | 20th century | Kühnel et al. (2017a) |
| Reptiles (Reptilia) | From 1920 on | 20th century | Kühnel et al. (2017b) |
| Fish and lampreys (Pisces et Cyclostomata) | Since 1787 | 18th century | SenUVK (2019) |
| Molluscs (Mollusca: Gastropoda und Bivalvia) | Since 1766 | 18th century | Hackenberg and Müller (2017) |
| Spiders (Araneae) and harvestmen (Opiliones) | Since the beginning 20th century | 20th century | Platen and Broen (2005) |
| Butterflies and moths (Lepidoptera: Makrolepidoptera) | Since 1772 | 18th century | Speyer and Speyer (1862) |
| Dragonflies (Odonata) | Since the middle of the 19th century | 19th century | Burmeister (1838); Sélys-Longchamps (1850; 1858); Stein (1863) |
| Grasshoppers and crickets (Saltatoria: Ensifera et Caelifera) | Since 1750 | 18th century | Machatzi et al. (2005) |
| Cicadas (Hemiptera: Fulgoromorpha und Cicadomorpha) | Since 1950 | 20th century | Strübing (1956) |
| Snakeflies, alderflies, dobsonflies, and lacewings (Raphidioptera, Megaloptera, Neuroptera) | After 1900 | 20th century | Enderlein (1906); Schirmer (1912); Wanach (1915) |
| Bees and wasps (Hymenoptera part.) with ants | Since 1856 | 19th century | Saure et al. (2005b) |
| Caddisflies (Trichoptera) | Before 1882 | 19th century | Mey (2005) |
| Earwingflies (Mecoptera) | Since 1912/1915 | 20th century | Schirmer (1912); Wanach (1915) |
| Robber flies (Diptera: Asilidae) | Since 1842 | 19th century | Degen (2017) |
| Mayflies (Ephemeroptera) | Since 1878 | 19th century | Müller (2017) |
| Hoverflies (Diptera: Syrphidae) | Since 1822 | 19th century | Wolf (1998) |
| Aquatic beetles (Coleoptera: Hydradephaga, Hydrophiloidea part., Hydraenidae, Elmidae und Dryopidae) | Since 1889 | 19th century | Hendrich and Müller, (2017) |
| Ground beetles (Coleoptera: Carabidae) | Since 1837 | 19th century | Erichson (1837; 1839) |
| Jewel beetles (Coleoptera: Buprestidae) | Before 1900 | 19th century | Gottwald (2017) |
| Leaf beetles (Chrysomelidae und Megalopodidae) | Before 1900 | 19th century | Heinig and Schöller (2017) |
| Scarab beetles (Coleoptera: Scarabaeoidea) | Since 1889 | 19th century | Esser (2017) |
| Long-horned beetles (Coleoptera: Cerambycidae) | Since 1919 | 20th century | Reineck (1919) |
| Weevils (Curculionoidea) | Since 1898 | 19th century | Bayer and Winkelmann (2004) |
| True bugs (Heteroptera) | Since the middle of the 19th century | 19th century | Deckert and Burghardt (2018) |
| Short-winged beetles and hister beetles (Coleoptera: Staphylinoidea und Histeridae) | Since 1935 | 20th century | Korge (2003) |
| Capuchin beetles (Bostrichoidea), multicoloured beetles (Cleroidea), flat beetles (Cucujoidea), click beetles (Elateroidea), shipyard beetles (Lymexyloidea) and black beetles (Tenebrioidea) | Before 1900 | 19th century | Esser (2017) |
| Bagmoths (Lepidoptera: Psychidae) | Since 1766 | 18th century | Weidlich (2022) |
